# Sesquiterpenoids Specially Produced by Fungi: Structures, Biological Activities, Chemical and Biosynthesis (2015–2020)

**DOI:** 10.3390/jof7121026

**Published:** 2021-11-30

**Authors:** Quan Dai, Fa-Lei Zhang, Tao Feng

**Affiliations:** School of Pharmaceutical Sciences, South-Central University for Nationalities, Wuhan 430074, China; quandai@mail.scuec.edu.cn (Q.D.); flzhang@mail.scuec.edu.cn (F.-L.Z.)

**Keywords:** sesquiterpenoids, fungus, structures, structural diversity, biological activity, synthesis

## Abstract

Fungi are widely distributed in the terrestrial environment, freshwater, and marine habitat. Only approximately 100,000 of these have been classified although there are about 5.1 million characteristic fungi all over the world. These eukaryotic microbes produce specialized metabolites and participate in a variety of ecological functions, such as quorum detection, chemical defense, allelopathy, and maintenance of symbiosis. Fungi therefore remain an important resource for the screening and discovery of biologically active natural products. Sesquiterpenoids are arguably the richest natural products from plants and micro-organisms. The rearrangement of the 15 high-ductility carbons gave rise to a large number of different skeletons. At the same time, abundant structural variations lead to a diversification of biological activity. This review examines the isolation, structural determination, bioactivities, and synthesis of sesquiterpenoids that were specially produced by fungi over the past five years (2015–2020).

## 1. Introduction

**Figure 1:**
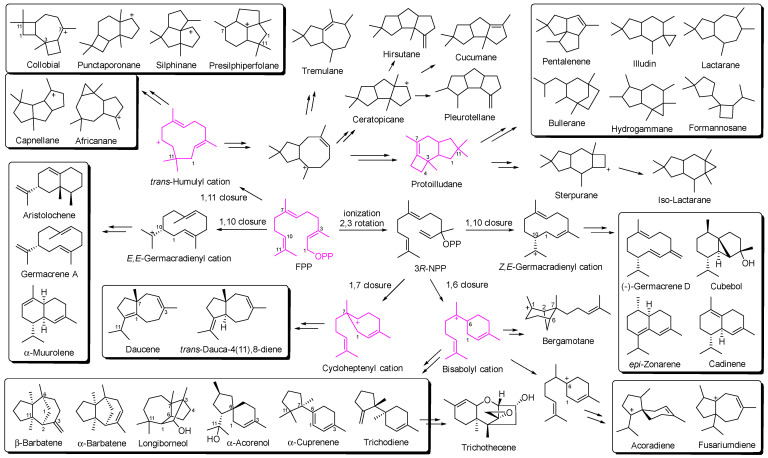
Cyclization of FPP by characterized fungal sesquiterpene synthases (Reference [3]).

Fungi are undoubtedly important resources for natural products discovery. With the advancement of natural product research, the importance of its biological resources has been infinitely enlarged. In the giant natural product system of fungi, sesquiterpenes, due to their carbon skeletons and amounts, are the largest of all types. The C-15-hydrocarbon skeletal system of various sesquiterpenoids isolated from fungi, bacteria, and plants are synthesized from farnesyl pyrophosphate (FPP) under the catalysis of sesquiterpene synthases [1,2]. Sesquiterpene synthases catalyze different initial cyclization reactions to produce secondary or tertiary cyclic carbocation intermediates, which can then be further cyclized and reassembled until carbocation quenching at the active center, followed by the enzymatic release of the final sesquiterpenoid scaffold (Figure 1) [3]. A huge number of sesquiterpenoids were, consequently, produced [4,5,6]. Among various other resources, fungal species have an enormous contribution owing to their potential to carry out the bio-transformations and drug synthesis under environmentally acceptable conditions. For instance, hydroxymethylacylfulvene (HMAF) is a semisynthetic antitumor agent based on the naturally occurring illudin S occurring in the mushroom *Omphalotus olearius* [7]. It has been advanced into human clinical trials for the treatment of cancers [8,9]. Trichothecenes, a class of tricyclic sesquiterpenes produced by a wide variety of fungi, are toxic to animals and humans and frequently present in cereal crops. They have attracted much attention in the areas such as agriculture, food contamination, and health care [10,11,12,13].

**Figure 2:**
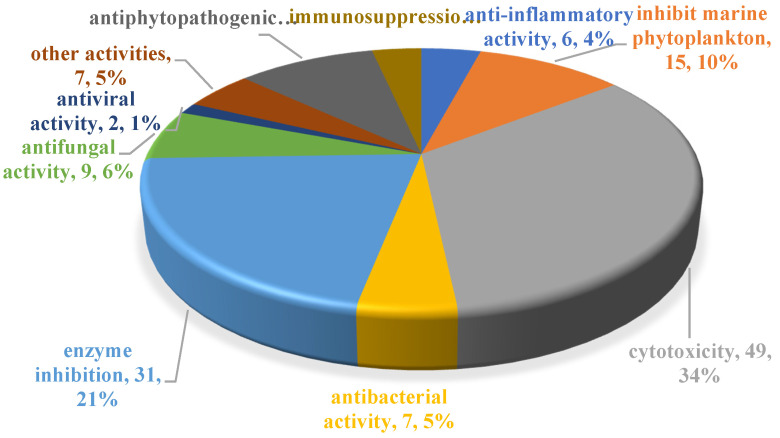
The proportion of one activity compared to the whole occurrence of activities of bioactive fungal sesquiterpenoids.

Our research group has been engaged in the study of the chemical composition of fungi for decades [14,15], while a large number of sesquiterpenoids have been reported [6]. It has been found that the vast majority of skeletons, such as alliacane, bergamotane, hirsutane, tremulane, etc., are specially produced by fungi. Many compounds displayed significant biological activities, and it is obvious that cytotoxic activity accounts for the largest proportion (Figure 2). In addition, with the development of synthetic biology, the biosynthesis of many fungal sesquiterpenoids has been figured out. This review gives an overview about the structures, biological activities, chemical synthesis and biosynthesis of sesquiterpenoids specially produced by fungi presented from 2015 to 2020.

## 2. Composition and Bioactivities

### 2.1. Alliacane, Cadinene, Azulene, and Zierane

Nine alliacane sesquiterpenoids inonoalliacanes A–I **1a**/**1b**–**6a**/**6b**–**7**–**9** were isolated from the culture broth of the basidiomycete *Inonotus* sp. BCC 22670 [16]. Inonoalliacane A **1** exhibited moderate antibacterial activity against *Bacillus cereus* with a minimum inhibitory concentration (MIC) value of 25 µg/mL. Inonoalliacane B **2** showed antiviral activity against herpes simplex virus type 1 (HSV-1) with IC_50_ of 17 μg/mL.

Clitocybulols G–O **10**–**18**, highly oxidized alliacane sesquiterpenoids, were isolated from the solid culture of the edible fungus *Pleurotus cystidiosus* [17]. Clitocybulols G **10** and L **15** showed weak inhibitory activity against protein tyrosine phosphatase-1B (PTP1B) with IC_50_ values of 49.5, 38.1 μM, respectively. 

In the ^1^H NMR-guided fractionation of extracts from the edible mushroom *Lactarius deliciosus*, two new azulene-type sesquiterpenoids **19** and **20** were characterized [18]. Pestabacillin A **21** bearing a zierane-type sesquiterpene skeleton was isolated from the co-culture of the endophytic fungus *Pestalotiopsis* sp. with *Bacillus subtilis* [19]. Furthermore, the absolute configuration of **21** was confirmed by single-crystal X-ray diffraction analysis.



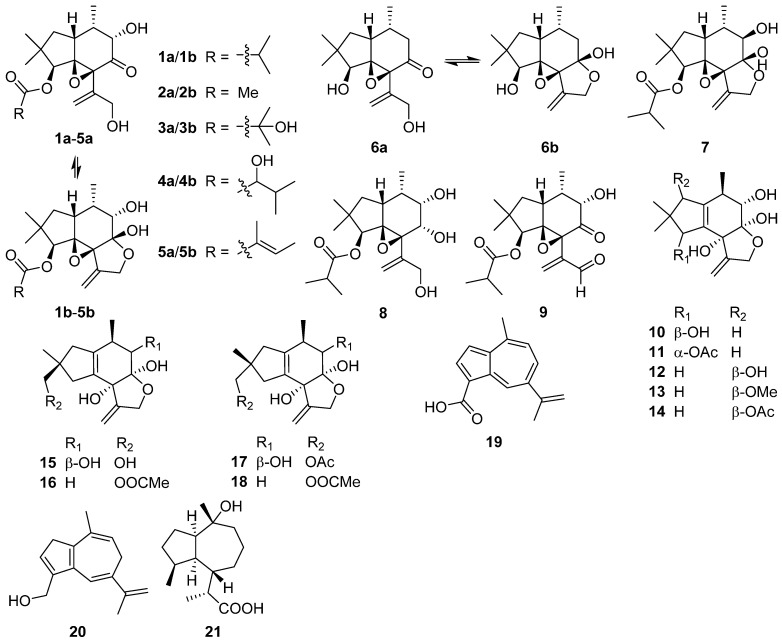



### 2.2. Bergamotane, Spiroaminal, and Spiroaxane

Bergamotane sesquiterpenes bearing a bridged 6/4 bicyclic ring incorporated with an isopentyl unit, are naturally occurring in plants and fungi [20,21]. A new class of polyoxygenated bergamotanes with notable features inspired by a 6/4/5/5 tetracyclic ring system was very rare in nature and all examples of the polycyclic bergamotanes only derived from fungi [22,23,24,25]. 



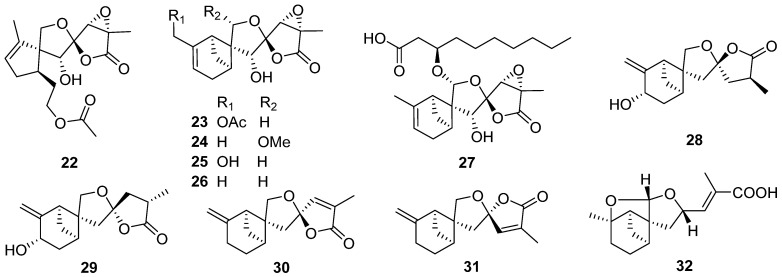



**Scheme 1:**
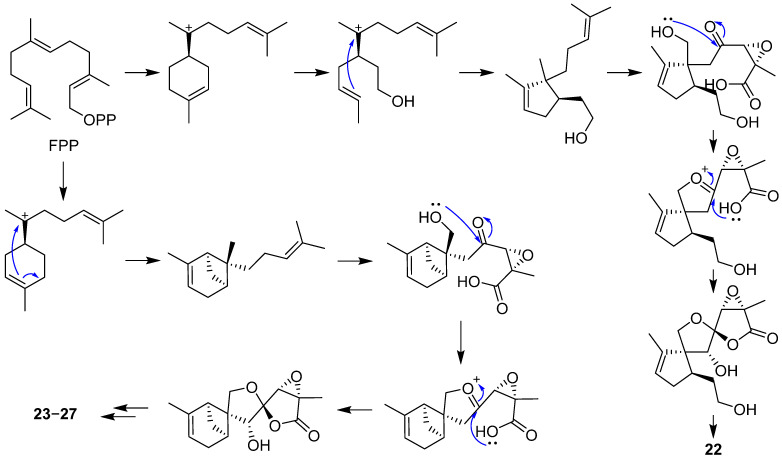
Plausible biogenetic pathways for **22**–**27** (Reference [26]).

Purpurolide A **22**, an unprecedented sesquiterpene lactone with a rarely encountered 5/5/5 spirocyclic skeleton, along with five new 6/4/5/5 tetracyclic sesquiterpene lactones (purpurolides B–F **23**–**27**), was isolated from the cultures of the endophytic fungus *Penicillium purpurogenum* [26,27]. The structures and absolute configurations of **22**–**27** were established by spectroscopic analysis, a single-crystal X-ray diffraction, and calculations of the ^13^C NMR and ECD data. The plausible biosynthetic pathway of **22**–**27** is shown in Scheme 1. Compounds **22**–**27** showed significant inhibitory activity against pancreatic lipase with IC_50_ values of 1.22–7.88 μM.



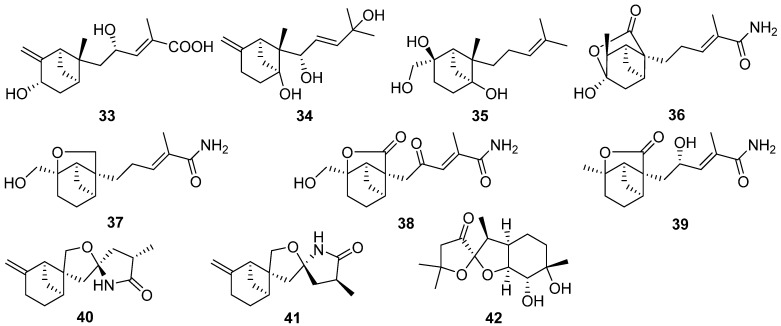



Expansolides C **28** and D **29** were two new bergamotane sesquiterpene lactones isolated from the plant pathogenic fungus *Penicillium expansum* [28]. The epimeric mixture of expansolides C **28** and D **29** (in a ratio of 2:1 at the temperature of the bioassay) exhibited more potent *α*-glucosidase inhibitory activity (IC_50_ 0.50 mM) as compared with the positive control acarbose (IC_50_ 1.90 mM) in an in vitro bioassay. 

Donacinolides A **30** and B **31** and donacinoic acids A **32** and B **33**, four new rare tetracyclic bergamotane-type sesquiterpenoids, were isolated from the mushroom-associated fungus *Montagnula donacina* [29]. Two new β-bergamotane sesquiterpenoids **34** and **35** were isolated from the marine-derived fungus *Aspergillus fumigatus* [30]. Brasilamides K–N **36**–**39** were isolated from the plant endophytic fungus *Paraconiothynium Brasiliense* [31].

**Scheme 2:**
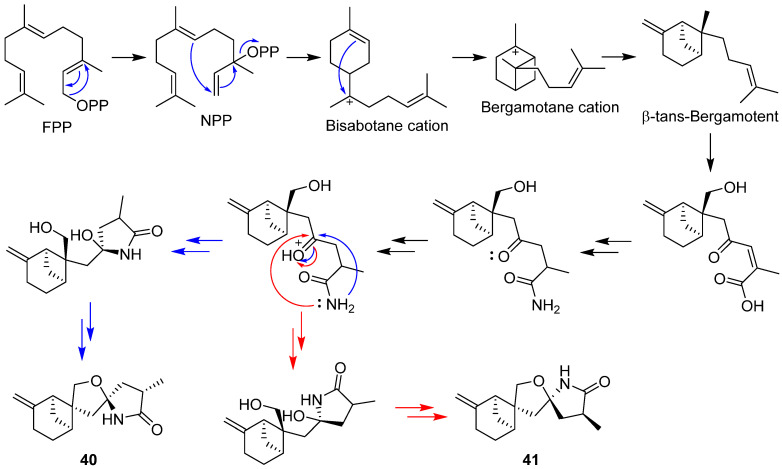
Plausible biosynthetic pathways of sporulaminals A **40** and B **41** (Reference [32]).

Sporulaminals A **40** and B **41**, a pair of unusual epimeric spiroaminal derivatives bearing a 6/4/5/5 tetracyclic ring system derived from bergamotane sesquiterpenoid (Scheme 2), were isolated from a marine-derived fungus *Paraconiothyrium sporulosum* [32]. Pleurospiroketal F **42**, a new perhydrobenzannulated 5,5-spiroketal sesquiterpene was isolated from solid-state fermentation of *Pleurotus citrinopileatus*, and the absolute configuration of **42** was determined by single-crystal X-ray diffraction analysis [33].

Flammuspirones A–J **43**–**52**, ten spiroaxane sesquiterpenoids, were obtained from the edible mushroom *Flammulina velutipes* [34]. Flammuspirones A **43** and C **45** showed inhibition on HMG-CoA reductase with IC_50_ of 114.7 and 77.6 μM, respectively. Flammuspirones C–E **45**–**47** and H **50** showed inhibitory activity on DPP-4 with IC_50_ values in the range from 70.9 to 83.7 μM. 

Talaminoid A **53** was obtained from the fungus *Talaromyces minioluteus* [35]. Talaminoid A **53** showed a significant suppressive effect on the production of nitric oxide (NO) on lipopolysaccharide (LPS) induced BV-2 cell, with IC_50_ of 5.79 μM. In addition, talaminoid A **53** exhibited significant anti-inflammatory activities against the production of TNF-*α* and IL-6. Further immunofluorescence experiments revealed the mechanism of action to be inhibitory the NF-κB-activated pathway. A new sesquiterpenoid **54** was isolated from the fungus *Pholiota nameko* [36]. Tramspiroins A–D **55**–**58** have been isolated from the cultures of Basidiomycete *Trametes versicolor* [37].



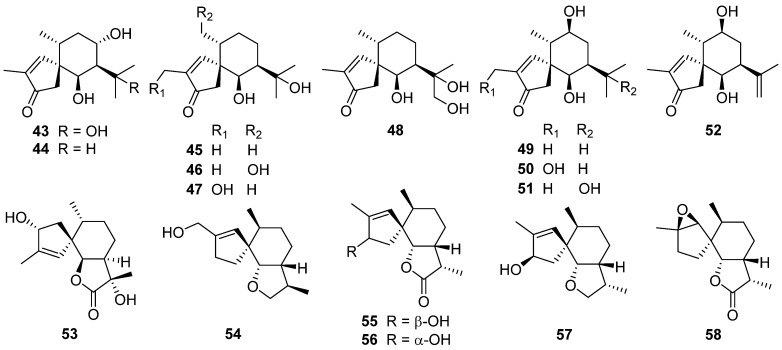



### 2.3. Carotane, Cyclonerane, Cyclofarnesane, and Longifolene

A new dimeric sesquiterpene divirensol H **59** and two exceptionally novel trimeric sesquiterpenes trivirensols A **60** and B **61** were purified from an endophytic fungus *Trichoderma virens* [38]. Divirensol H **59** showed significant activities against fungi *Penicillium italicum*, *Fusarium oxysporum*, *Fusarium graminearum*, *Colletotrichum musae*, and *Colletotrictum gloeosporioides* with MIC values of 6.25 to 25 μg/mL. Rhinomilisin A **62** and four new heptelidic acid derivatives, rhinomilisin B–E **63**–**66**, were isolated from the endophytic fungus *Rhinocladiella similis* [39]. Rhinomilisins A **62** showed moderate cytotoxicity activity against the mouse lymphoma cell line L5178Y with an IC_50_ value of 5.0 μM.



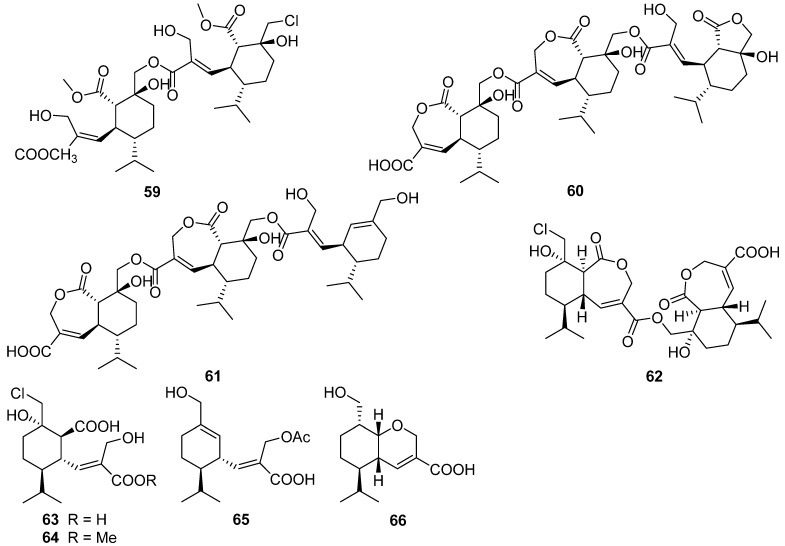





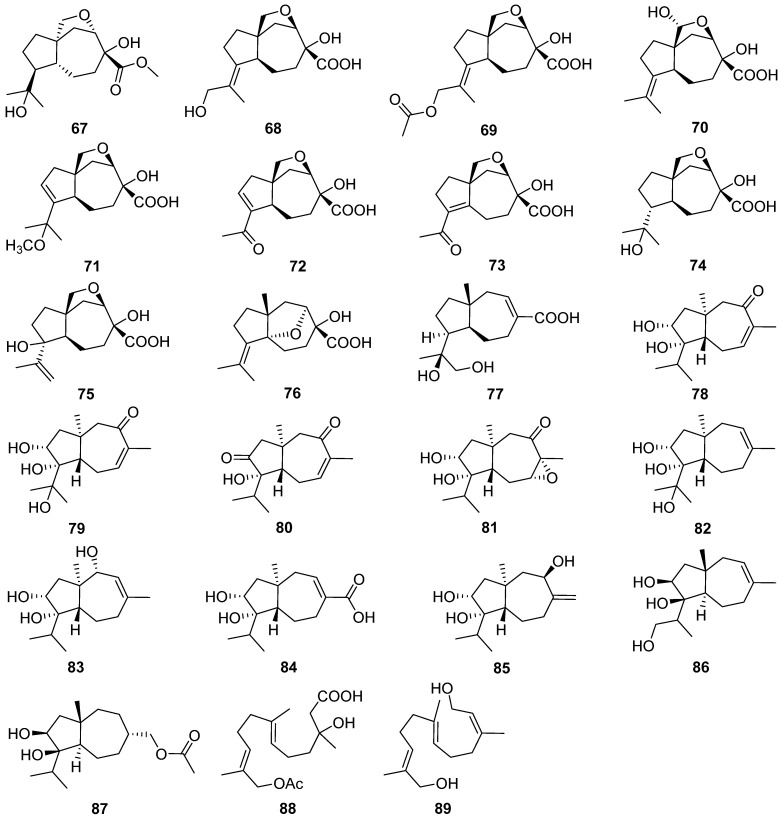



Peniterester **67**, a new tricyclic sesquiterpene was isolated from the secondary metabolites of an artificial mutant *Penicillium* sp. T2-M20 [40]. Peniterester **67** showed significant activities against *Bacillus subtilis*, *Escherichia coli*, and *Staphylococcus aureus* in vitro with MICs of 8.0, 8.0, and 4.0 μg/mL, respectively. 

Piltunines A–F **68**–**73** and penigrisacids A–D **74**–**77**, ten new carotane sesquiterpenoids, were isolated from the marine-derived fungus *Penicillium griseofulvum* and *Penicillium piltunense*, respectively [41,42]. Penigrisacid D **75** showed a weak effect on ECA-109 tumor cells with an IC_50_ value of 28.7 µM [41]. Trichocarotins A–H **78**–**85**, eight new carotane sesquiterpenes, were isolated from the culture of the fungus *Trichoderma virens* [43]. Trichocarotins C–E **80**–**82** and H **85** displayed potent inhibition against the four marine phytoplankton species (*Chattonella marina*, *Heterosigma akashiwo*, *Karlodinium veneficum*, and *Prorocentrum donghaiense*) tested, especially against *C. marina* with IC_50_ values ranging from 0.24 to 1.2 μg/mL.

Trichocaranes E **86** and F **87** were isolated from cultures of the insect pathogenic fungus *Isaria fumosorosea* [44]. Trichocaranes E **86** and F **87** showed potent cytotoxic activities against six tumor cell lines MDA, MCF-7, SKOV-3, Hela, A549, and HepG2 with IC_50_ values in a concentration range of 0.13–4.57 μg/mL. Two new carotane-type biogenetically related sesquiterpenes, aspterrics A **88** and B **89**, were isolated from the deep-sea-derived fungus *Aspergillus terreus* [45].



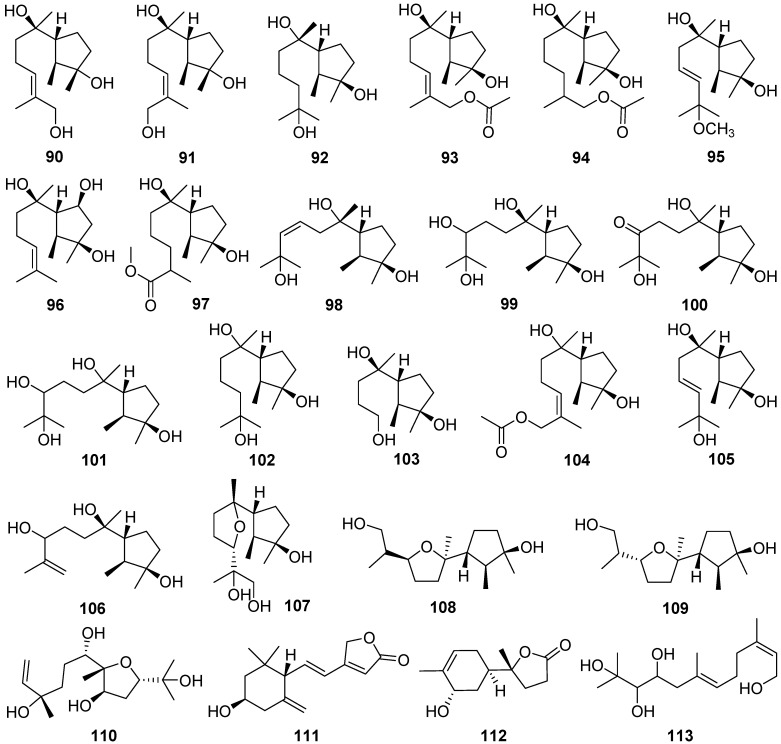



Two new cycloneranes **90** and **91** were isolated from the marine alga endophytic fungus *Trichoderma citrinoviride* [46]. The compound **90** had an inhibition to the marine phytoplankton species *Karlodinium veneficum* with an IC_50_ value of 8.1 μg/mL. Six new cycloneranes **92**–**97** were isolated from the fungus *Trichoderma harzianum* [47,48,49]. The three new ones **95**–**97** all exhibited growth inhibition of the four phytoplankton species (*Chattonella marina*, *Heterosigma akashiwo*, *Karlodinium veneficum*, and *Prorocentrum donghaiense*) with IC_50_ values ranging from 0.66 to 75 μg/mL [49]. 

Cyclonerotriol B **98** was isolated from the soil fungus *Fusarium avenaceum* [50]. Cyclonerodiol B **99** was isolated from the mangrove plant endophytic fungus *Trichoderma* sp. Xy24 [51]. Cyclonerodiol B **99** exhibited significant neural anti-inflammatory activity by inhibiting LPS-induced NO production in BV2 cells with the inhibitory rates of 75.0% at 0.1 μM, which are more potent than curcumin, positive control with the inhibitory rate of 21.1% at 0.1 μM. 



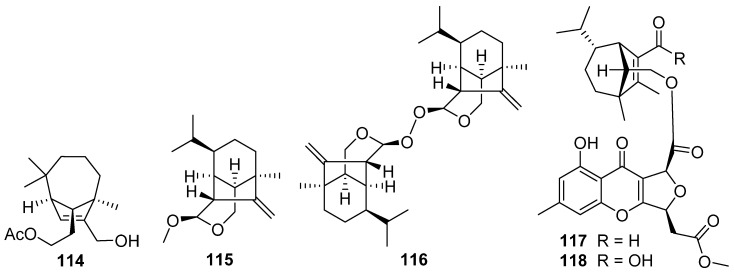



Ten new cycloneranes **100**–**109** were isolated from the algicolous endophytic fungus *Trichoderma asperellum* [52,53]. The seven new ones, **100**–**104**, **108**, and **109**, all exhibited growth inhibition of the four phytoplankton species (*Chattonella marina*, *Heterosigma akashiwo*, *Karlodinium veneficum*, and *Prorocentrum donghaiense*) with IC_50_ values ranging from 2.4 to 76 μg/mL [52].

A new sesquiterpenoid **110** was isolated and identified from an endophytic fungus *Umbelopsis dimorpha* grown on host-plant *Kadsura angustifolia* and wheat bran [54]. Inonofarnesane **111**, a new cyclofarnesane sesquiterpenoid, was isolated from cultures of the wood-rotting basidiomycete *Inonotus* sp. BCC 23706 [55]. 

One new norbisabolane sesquiterpenoid degradation, isopolisin B **112**, was isolated from the fungus *Pestalotiopsis heterocornis* [56]. Koninginol D **113** as a new farnesane sesquiterpenoid was isolated from the endophytic fungus *Trichoderma koningiopsis* [57].

Bipolenin F **114**, a new *seco*-longifolene sesquiterpenoid, and two new *seco*-sativene sesquiterpenoids, bipolenins D **115** and E **116**, and two novel sesquiterpenoid-xanthone adducts, bipolenins I **117** and J **118**, were obtained from cultures of potato endophytic fungus *Bipolaris eleusines* [58,59]. Bipolenins I **117** and J **118** exhibited potent inhibitory activity against the plant pathogens *Alternaria solani* with MIC values of 8 and 16 μg/mL, respectively [59].



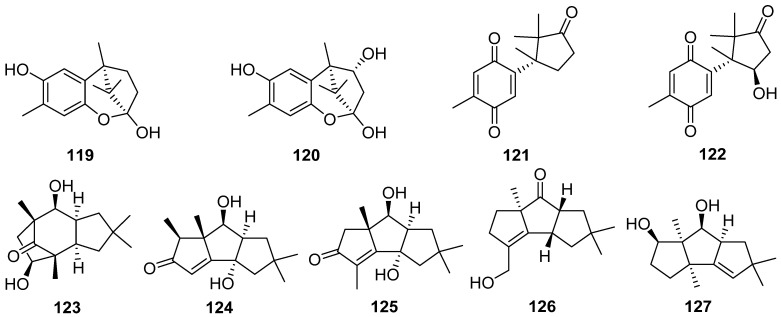



### 2.4. Cerapicane, Cucumane, Cuparene, Hirsutane, Isohirsutane, and Triquinane

Cuparane-type sesquiterpenoids of fungal origin possess a skeleton with a six-membered ring connected to a five-membered ring, of which the six-membered ring is always aromatic. Linear triquinane sesquiterpenoids have a basic skeleton 1*H*-cyclopenta[α]pentalene [60]. Many compounds displayed a wide range of biological activities, such as cytotoxic, antimicrobial, and anti-inflammatory activities. A review gives an overview about the isolation, structure, biological activities, and chemical synthesis of linear triquinane sesquiterpenoids [61]. 



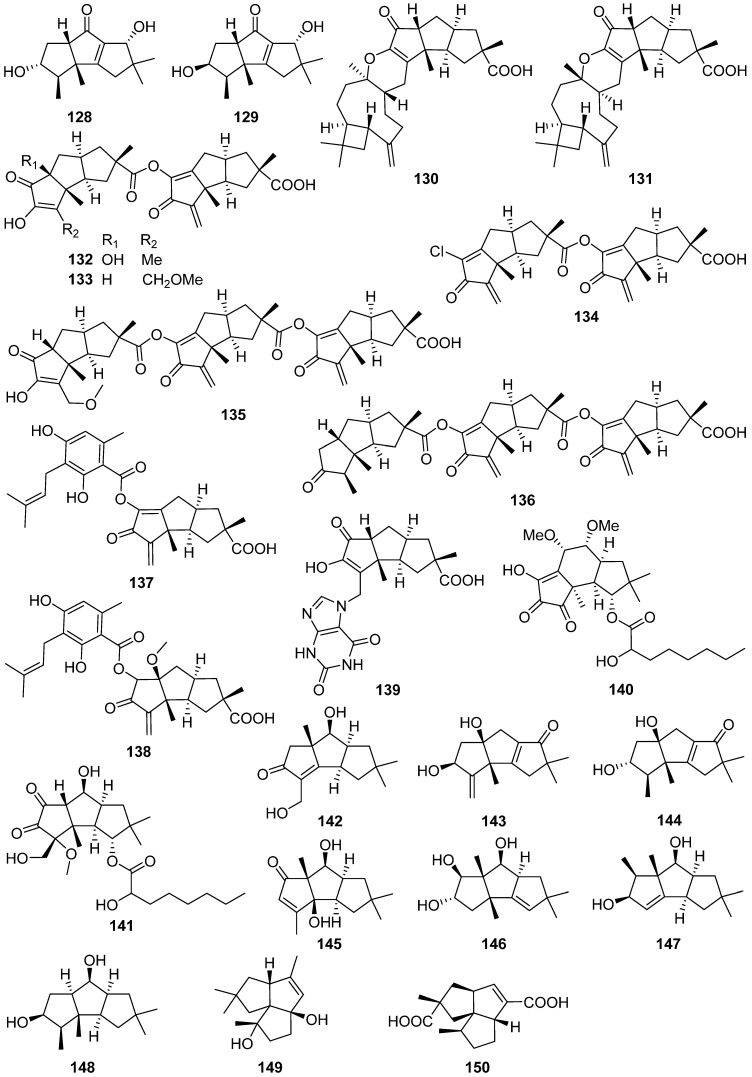



Enokipodins A–D **119**–**122**, highly oxygenated cuparene-type sesquiterpenes were obtained from the fungi *Flammulina rossica* and *Flammulina velutipes*. In addition, enokipodins B **121** and D **122** are oxidized compounds of enokipodins A **119** and C **120**, respectively [62]. 

One new cerapicane cerrenin A **123**, and two new isohirsutane sesquiterpenoids cerrenins B **124** and C **125**, were isolated from the broth extract of *Cerrena* sp. which was isolated from *Pogostemon cablin* [63]. Trefoliol C **126**, one new cucumane sesquiterpenoid, was isolated from cultures of the basidiomycetes *Tremella foliacea* [64]. A new sesquiterpenoid **127** was isolated from the crude extract of *Antrodiella albocinnamomea* [65]. Two new hirsutane-type sesquiterpenoids, chondrosterins N **128** and O **129**, were isolated from the marine fungus *Chondrostereum* sp. [66]. 

Ten new hirsutane-type sesquiterpenoids, sterhirsutins C–L **130**–**139**, were isolated from the culture of *Stereum hirsutum* [67]. Sterhirsutins C **130** and D **131** possessed an unprecedented chemical skeleton with a 5/5/5/6/9/4 fused ring system, and the absolute configuration of sterhirsutin C **130** was assigned by single-crystal X-ray diffraction experiment. Sterhirsutin L **139** was the first sesquiterpene coupled with a xanthine moiety. Sterhirsutins C–L **130**–**139** showed cytotoxicity against K562 and HCT116 cell lines, and sterhirsutin K **138** induced autophagy in HeLa cells. Sterhirsutin G **133** inhibited the activation of the IFNβ promoter in Sendai virus-infected cells.

Cerrenins D **140** and E **141**, two new triquinane-type sesquiterpenoids, were obtained from the endophytic fungus *Cerrena* sp. A593 [68]. Chondrosterins K–M **142**–**144** were isolated from the marine fungus *Chondrostereum* sp. [69]. Chondrosterins K–M **142**–**144** showed different degrees of cytotoxicities against various cancer cell lines (CNE1, CNE2, HONE1, SUNE1, A549, GLC82, and HL7702) in vitro, with IC_50_ values ranging from 12.03 to 58.83 µM.



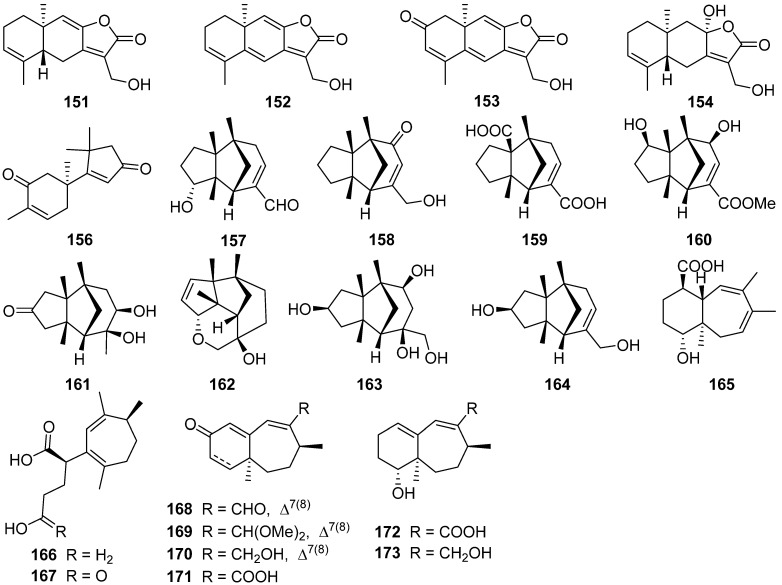



Antrodins A–E **145**–**149** were isolated from the fermentation of *Antrodiella albocinnamomea* [70]. Tremutin H **150** was isolated from cultures of the basidiomycetes *Irpex lacteus* [71]. The absolute configuration of **150** was determined by single-crystal X-ray diffraction analysis, and **150** shows a weak inhibitory effect on NO production with an IC_50_ value of 22.7 μM.

### 2.5. Eudesmanolide, Gymnomitrane, and Humulane

Humulane-type sesquiterpenoids are found rarely in nature. They have been recognized as being biogenetic precursors of many types of sesquiterpenoids [6]. The macrocyclic nature of members of the humulane group has proved to be troublesome for the determination of their absolute configurations.

**Scheme 3:**
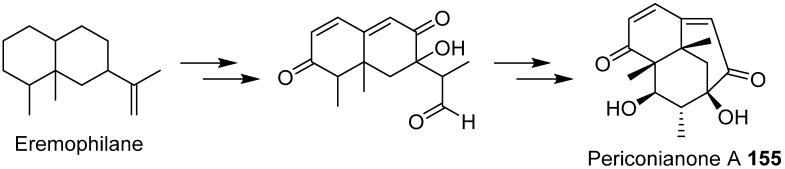
Hypothetical biosynthetic pathway of periconianone A **155** (Reference [73]).

**Scheme 4:**
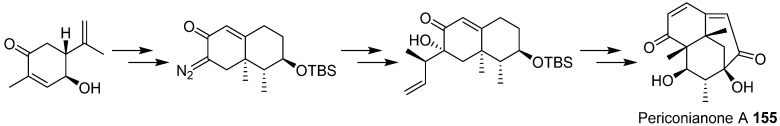
Total synthesis of periconianone A **155** (Reference [74]).

Four new 12,8-eudesmanolides **151**–**154** were isolated from a mangrove rhizosphere-derived fungus *Eutypella* sp. 1–15 [72]. Periconianone A **155**, a polyoxygenated sesquiterpenoid with a new 6/6/6 tricarbocyclic skeleton, was isolated from the endophytic fungus *Periconia* sp., and the biosynthesis of the unusual six-membered carbonic ring of **155** was postulated to be formed through intramolecular aldol condensation (Scheme 3) [73]. The first enantioselective total synthesis of the periconianone A **155** based on a postulated biogenesis has been reported (Scheme 4) [74]. 

**Scheme 5:**
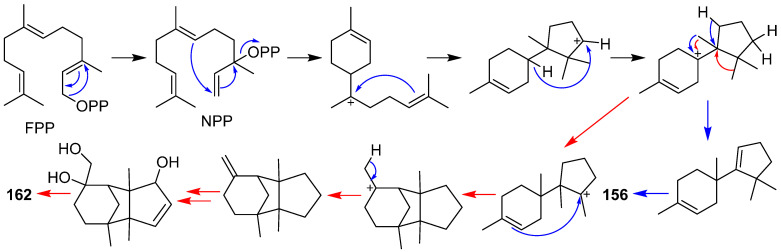
Proposed biosynthetic pathway of **156** and **162** (Reference [75]).

An unusual type sesquiterpene **156** possessed an unusual 14(7-6)-cuparane scaffold (Scheme 5), and six rarely-encountered gymnomitrane-type sesquiterpenoids **157**–**162**, were isolated from the medicinal mushroom *Ganoderma lingzhi* [75]. A new gymnomitrane-type sesquiterpenoid **163** was isolated from the fruiting body of *Ganoderma lucidum* [76]. This compound **163** significantly inhibited the growth of epidermal growth factor receptor-tyrosine kinase inhibitor EGFR-TKI-resistant human lung cancer A549 and human prostate cancer PC3 cell lines. Antrodin F **164** was isolated from the fermentation of *Antrodiella albocinnamomea* [70].

**Scheme 6:**
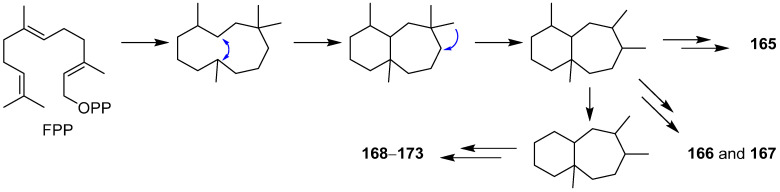
Postulated biogenetic pathway for ochracenes A–I **165–173** (Reference [77]).

Nine new humulane-derived sesquiterpenoids, ochracenes A–I **165**–**173**, were isolated from the Antarctic fungus *Aspergillus ochraceopetaliformis* [77]. A biogenetic pathway for them was given in Scheme 6. The two unprecedented 8,9-secocyclic sesquiterpenoids, ochracenes B **166** and C **167**, exhibited inhibitory effects on LPS-induced NO release in RAW 264.7 mouse macrophage cell with IC_50_ values of 14.6 and 18.3 μM, respectively.



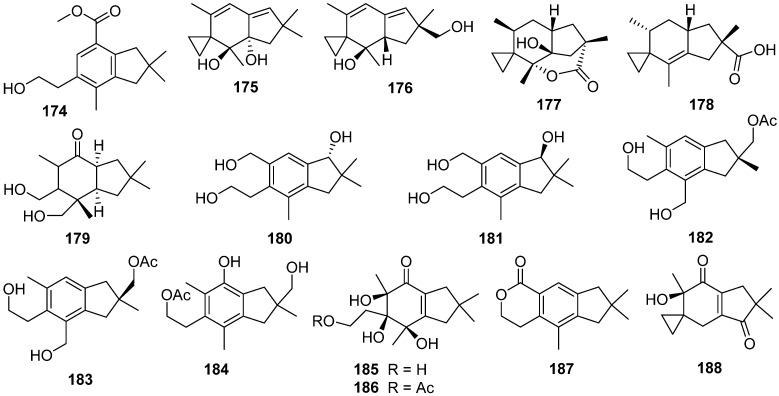



### 2.6. Illudane, Illudalane, Protoilludane, Marasmane, and Norilludane

A review offers a comprehensive description of the investigations that started with the discovery of illudins in 1950, led to HMAF clinical trials against various tumors as a single agent and in combination therapy beginning in 2002, and culminated in the past decade of advances in chemical synthesis and mechanisms of toxicity of AFs, including biotransformation processes, DNA alkylation products, unique influences of DNA repair capacities, and enzyme inhibition properties [9]. The 4/6/5 ring-fused protoilludane-type sesquiterpenoids are the precursors of many other sesquiterpenoids, representing the largest group of sesquiterpene metabolites of fungal origin.

Phellinignin D **174** was isolated from the fungus *Phellinus igniarius*, which possessed a new carbon skeleton that might derive from an illudane framework by methyl shift and aromatization [78]. Phellinignin D **174** showed moderate cytotoxicities to three human cancer cell lines (HL-60, SMMC-7721, and SW480) with the IC_50_ values of 21.1, 12.3, and 13.9 µM, respectively.

Illudadienes A **175** and B **176** were obtained from the wood-decomposing fungus *Granulobasidium vellereum* [79]. Phellinuin J **177** and sulphureuine A **178** were isolated from cultures of *Phellinus tuberculosus* and *Laetiporus sulphureus* [80]. Agrocybins H–K **179**–**184** were obtained from the edible mushroom *Agrocybe salicacola* [81]. Craterellins D **185** and E **186** were isolated from cultures of *Craterellus cornucopioides* [82]. Illudalane derivative, granulolactone **187**, and a 15-norilludane, granulodione **188**, were isolated from an agar plate culture of *Granulobasidium vellereum* [83]. 



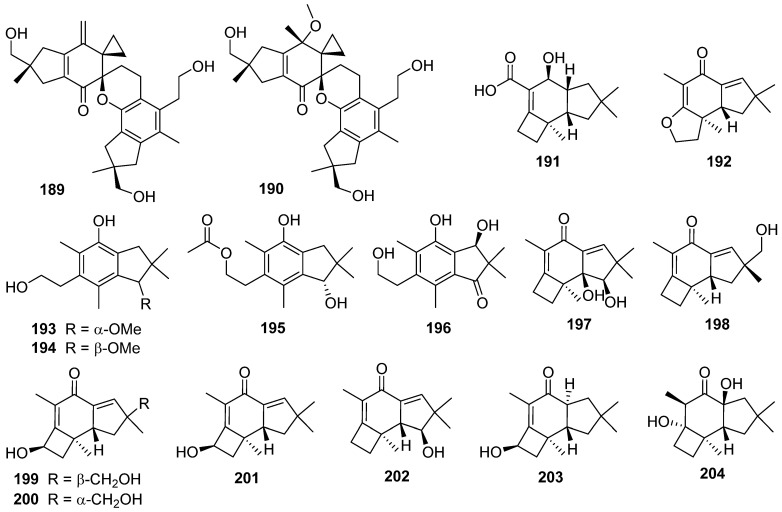



Two new disesquiterpenoid derivatives, bovistol B **189** and C **190**, and a new protoilludane derivative, pasteurestin C **191**, were isolated from the fermentation broth of the edible mushroom *Cyclocybe aegerita* [84]. Four illudalanes **192**–**195**, an unusual 2,3-*seco*-protoilludane **196**, and eight protoilludanes **197**–**204** were identified from the liquid culture of the endophytic fungus *Phomopsis* sp. TJ507A [85]. Phomophyllins A–G **196**–**202**, and phomophyllin I **204** displayed β-site amyloid precursor protein cleaving enzyme 1 (BACE1) inhibitory activities ranging from 19.4% to 43.8% at the concentration of 40 μM.

Epicoterpenes A–E **205**–**209**, and armilliphatic A **210** were isolated from *Armillaria* sp. by co-culture with the endophytic fungus *Epicoccum* sp. associated with *Gastrodia elata* [86]. Epicoterpene D **208** was the first example of an *ent*-protoilludane sesquiterpenoid scaffold bearing a five-membered lactone. Two new protoilludane sesquiterpene aryl esters **211** and **212** were isolated from the mycelium of *Armillaria mellea* [87]. Compound **212** showed cytotoxic activity for HepG2 cells with an IC_50_ value of 18.03 μg/mL. Three new sesquiterpene aryl esters, melleolide N **213**, Q **214**, and R **215**, were isolated from the EtOH extract of the mycelium of *Armillaria mellea* [88]. And **213**–**215** showed cytotoxicity to several human cancer cell lines.



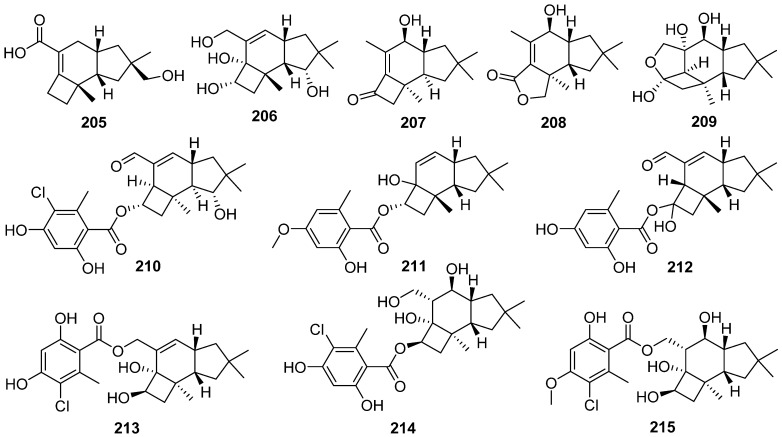



**Scheme 7:**
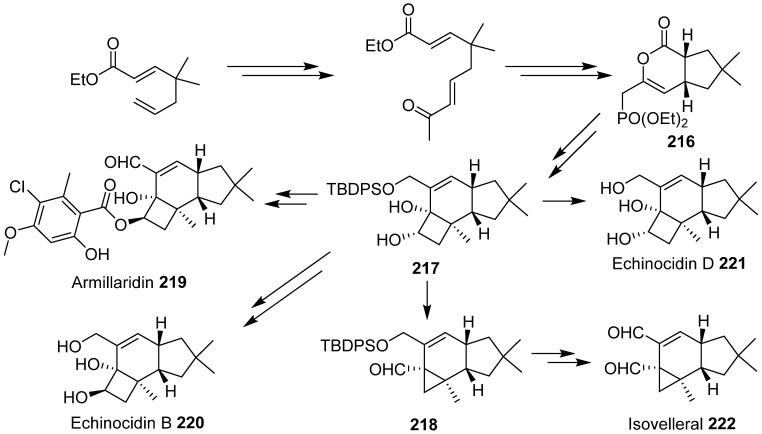
Synthesis of armillaridin **219**, echinocidins B **220** and D **221**, and isovelleral **222** (Reference [89]).

Unified total syntheses of marasmane, mellolide, and protoilludane sesquiterpenoids have been achieved through a key organocatalytic enantioselective annulation (Scheme 7) [89]. The elaboration of key bicyclic lactone **216** was the molecular springboard from which the first enantioselective total syntheses of protoilludanes echinocidin B **220** and echinocidin D **221**, and the mellolide armillaridin **219**, as well as the synthesis of the marasmane isovelleral **222**, were accomplished. The vanadium(II)/zinc(II) reductive coupling yielded the final ring of the densely functionalized *cis*-fused carbocyclic core. Finally, the unexpected semi-Pinacol-type ring contraction to establish cyclopropyl aldehyde **218** from cyclobutanediol **217** was potentially biomimetic in origin.



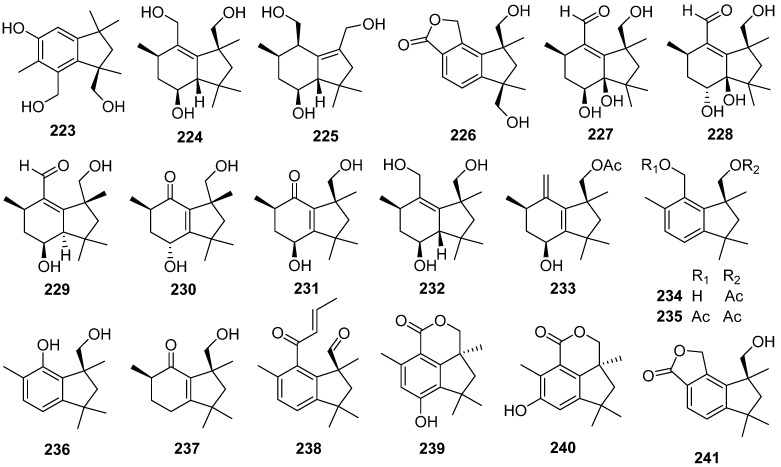





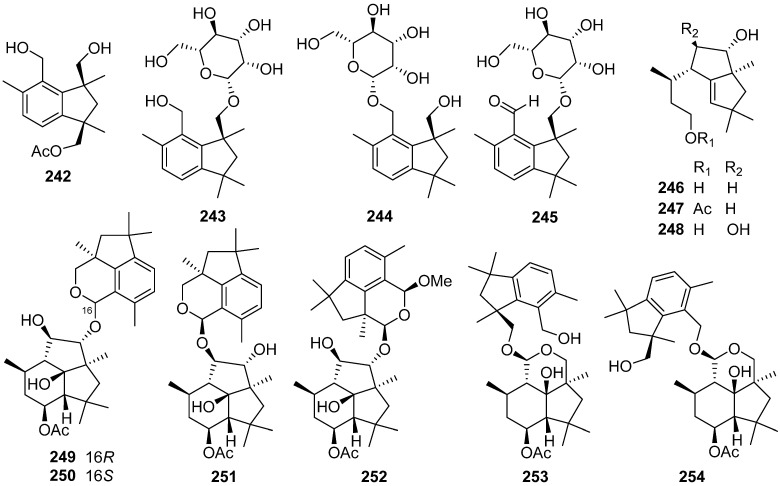



### 2.7. Botryane and Seco-Probotryane

A botryane-type sesquiterpenoid **223** was identified from the liquid culture of the endophytic fungus *Phomopsis* sp. TJ507A [85]. Arthrinins E–G **224**–**226**, three new sesquiterpenoids possessing non-isoprenoid botryane skeleton, were isolated from the endophytic fungus *Arthrinium* sp. HS66 [90]. Five new botryanes **227**–**231** were obtained from an endophytic fungus *Nemania bipapillata* [91]. Five new botryanes **232**–**236** were isolated from *Trichoderma oligosporum* [92]. Compounds **236** showed moderate cytotoxicity activity against K562 cells with an inhibitory rate of 45–60% at 6.25 µM (Taxol was used as a positive control with 60.3% inhibition at 2.0 µM). 

**Scheme 8:**
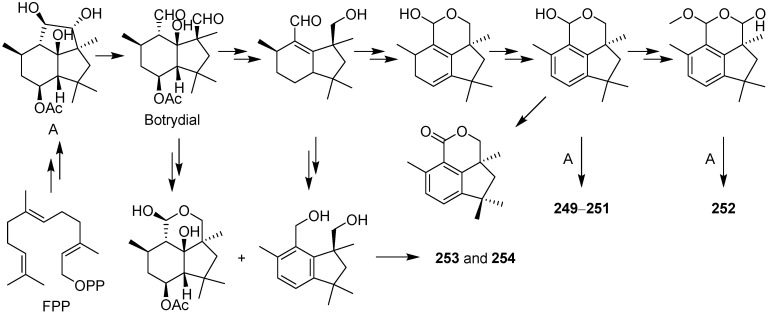
Plausible biosynthetic pathways for hypocriols A–F **249**–**254** (Reference [96]).

A new 10-norbotryane derivative **237** and three new botryanes **238**–**240** were isolated from the ascomycete *Hypoxylon rickii* [93,94]. Five new botryanes **241**–**245**, along with 4,5-*seco*-Probotryenols A–C **246**–**248** derived from cleavage of the probotryane skeleton at C-4/C-5, were isolated from *Stachybotrys bisbyi* [95]. Six new heterodimeric botryane ethers, hypocriols A–F **249**–**254**, were isolated from the insect-associated *Hypocrea* sp. EC1-35 [96]. A plausible biosynthetic pathway for **249**–**254** was given (Scheme 8). Hypocriols A–D **249**–**252** and F **254** showed significant activity against the HeLa cell, with IC_50_ values of 7.7, 3.1, 11.8, 3.8, and 4.6 μM, respectively. Hypocriol F **254** inhibited the proliferation of the HCT116 cell, showing an IC_50_ value of 2.7 μM.



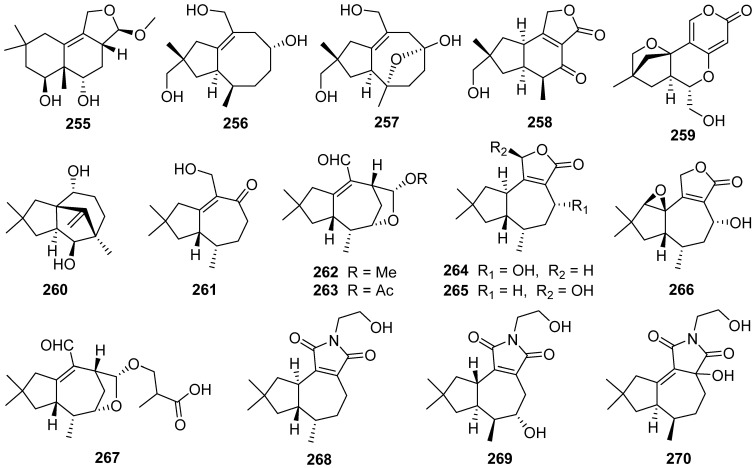



### 2.8. Tremulane, Sterpurane, Phlebiane, Merulane, and Irlactane

Tremulane-type sesquiterpenoids are a class of sesquiterpenoids with a 5/7-ringfused perhydroazulene carbon skeleton. The first example was isolated from the wood-decaying fungus *Phellinus tremulae* in 1993 [97]. The biosynthesis pathway was elucidated through a ^13^C-labeled feeding experiment revealed that tremulanes are derived from *trans*,*trans*-farnesyl pyrophosphate via humulene and a key step of methyl migration [98].

**Scheme 9:**
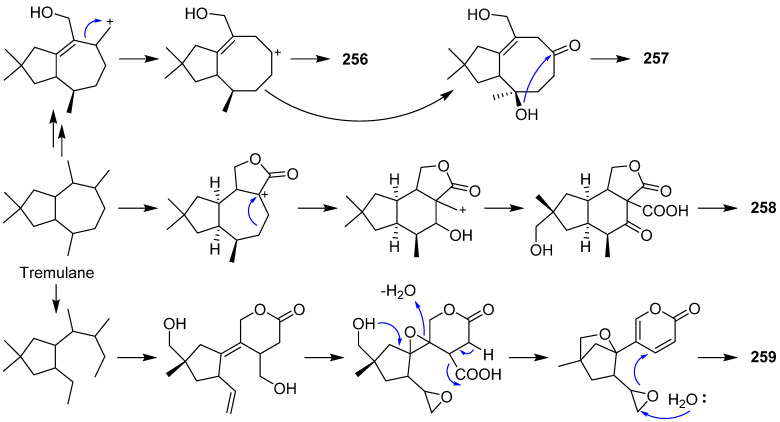
Proposed biosynthetic pathway for conosiligins A–D **256**–**259** (Reference [101]).

A new irlactane-type, irlactin K **255**, was isolated from the fermentation broth of the medicinal fungus *Irpex lacteus* [99]. The absolute configuration of **255** was established by single-crystal X-ray diffraction analysis. Irlactin K **255** could be derived from the tremulane type sesquiterpene irlactin E via a ring rearrangement [100]. Conosiligins A–D **256**–**259**, four ring-rearranged sesquiterpenoids, were isolated from cultures of the basidiomycete *Conocybe siliginea* [101]. Conosiligins A **256** and B **257** possessed a 5/8-fused ring system, while conosiligin C **258** has a 5/6-fused backbone conjugated with a γ-lactone. Conosiligin D **259** was a 5,6-seco tremulane derivative with the loss of a skeletal carbon, featuring a tetracyclic system involving a pyranone moiety (Scheme 9). Conosiligins C **258** and D **259** inhibited Con A-induced T cell proliferation with IC_50_ values of 12.3 and 6.6 μM, respectively.

**Scheme 10:**
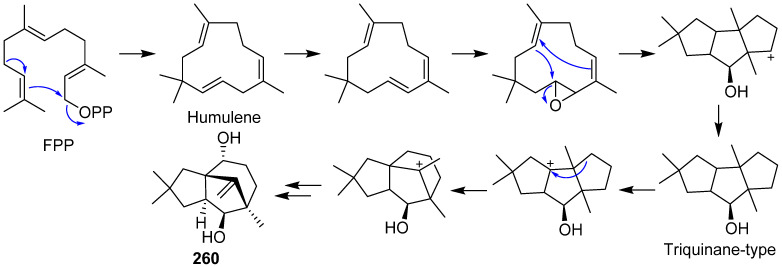
Proposed biosynthetic pathway for antroalbocin A **260** (Reference [102]).

Antroalbocin A **260** possessing a bridged tricyclic system was isolated from cultures of the higher fungus *Antrodiella albocinnamomea* [102]. The structure with the absolute configuration was determined by extensive spectroscopic methods and single-crystal X-ray diffraction analysis and a plausible biosynthetic pathway for **260** was proposed (Scheme 10).

Twenty-two tremulanes, irlactins F–J **261**–**265**, L–M **266**–**267**, irlactam A **268**, and irpexolactins A–N **269**–**282**, were isolated from cultures of the medicinal fungus *Irpex lacteus* [99,103,104,105]. Irlactin I **264** exhibited moderate cytotoxicities on HL-60, SMMC-7721, A-549, MCF-7, and SW480 cells with IC_50_ values of 16.23, 20.40, 25.55, 19.05, and 18.58 μM, respectively [104].



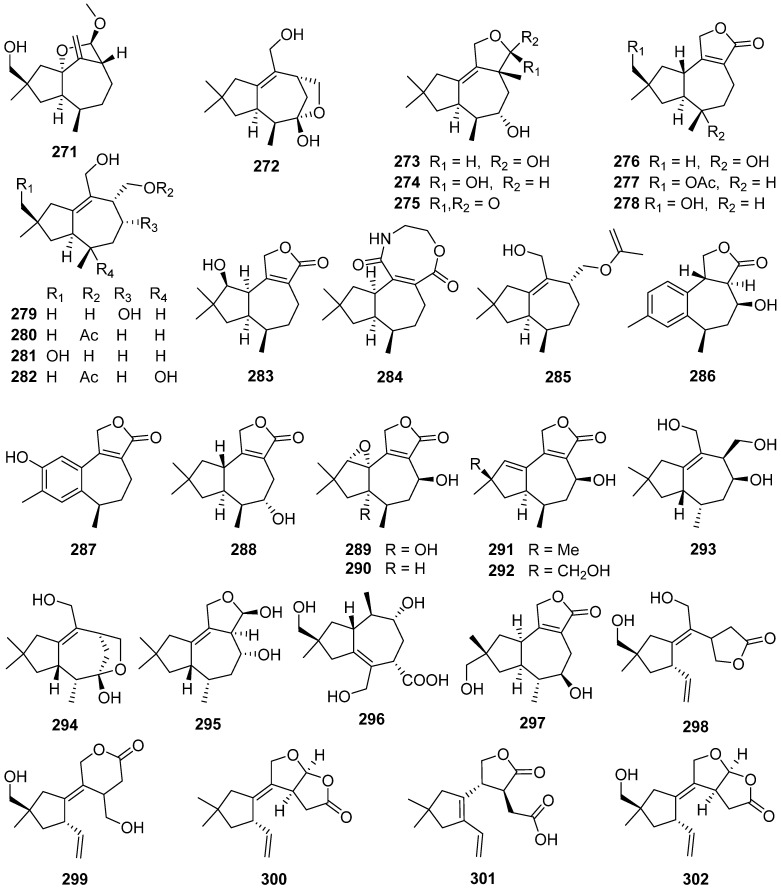





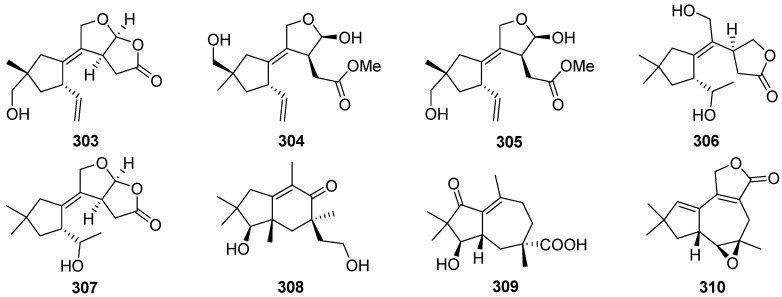



**Scheme 11:**
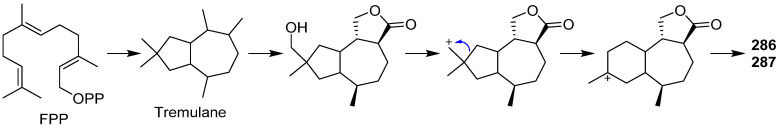
Proposed biosynthetic pathway for tremutins A **286** and B **287** (Reference [71]).

Phellinignins A–C **283**–**285** were new tremulane sesquiterpenoids that have been isolated from *Phellinus igniarius* [78]. Phellinignins A **283** and B **284** showed certain cytotoxicities to three human cancer cell lines (HL-60, SMMC-7721, and SW480) with the IC_50_ values of 0.7–17.4 µM, respectively. Tremutins A–G **286**–**292** were isolated from cultures of the basidiomycetes *Irpex lacteus* [71]. Tremutins A **286** and B **287** possessed an unusual 6/7-fused ring system that might be derived from a tremulane framework (Scheme 11), **289** and **290** were the first tremulane examples with a 1,2-epoxy moiety to be reported. Tremutin A **286** inhibited the lipopolysaccharide (LPS)-induced proliferation of B lymphocyte cells with an IC_50_ value of 22.4 μM. Tremutin B **287** inhibited concanavalin A (Con A)-induced T cell proliferation and LPS-induced B lymphocyte cell proliferation with IC_50_ values of 16.7 and 13.6 μM, respectively.

Nigrosirpexin A **293** was produced by *Nigrospora oryzae* co-cultured with *Irpex lacteus* [106]. Two new tremulanes **294** and **295** were obtained from different cocultures of *Nigrospora oryzae* and *Irpex lacteus* in a solid medium [107]. 5-Demethyl conocenol C **294** showed antifungal activities against *Didymella glomerate* and *Colletotrichum gloeosporioides* with MICs of 1 and 8 μg/mL, respectively. 

Davotremulanes A–D **296**–**299** were isolated from a plant-associated fungus X1-2 [108]. Davotremulanes A **296** and B **297** displayed selectively moderate activities to the A549 cell line with IC_50_ at 15.3, 25.2 μg/mL. A new tremulane sesquiterpenoid analogue **300** was isolated from the cultures of endophytic fungus *Colletotrichum capsica* [109]. Leptosphin B **301** was isolated from the endophytic fungus *Leptosphaeria* sp. XL026 [110]. Leptosphin B **301** showed moderate antibacterial activity against *Bacillus cereus* with a MIC value of 12.5 μg/mL. 

**Scheme 12:**
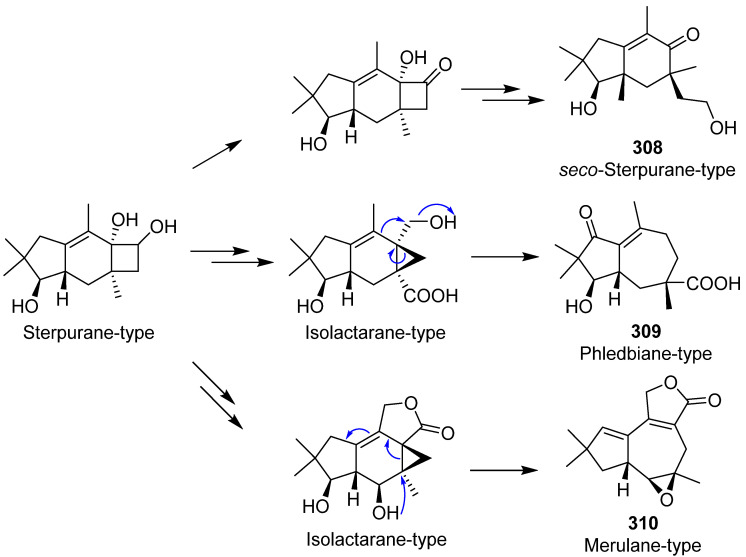
Plausible biogenetic pathways of **308**–**310** (Reference [112]).

Six 5,6-*seco*-tremulane analogues **302**–**307** were isolated from the culture broth of the medicinal fungus *Irpex lacteus* [111]. Two sesquiterpenes with new carbon skeletons, *seco*-sterpurane **308** and phlebiane **309**, and a novel merulane sesquiterpene **310** were isolated from cultures of the basidiomycete *Phlebia tremellosa* [112]. The plausible biogenetic pathways of **309** and **310** is shown in Scheme 12.

### 2.9. Trichothecene, Merosesquiterpenoid, Norsesquiterpenoid, and Pyrone

Trichothecenes are a family of sesquiterpenoid mycotoxins produced by multiple genera of fungi, including plant and insect pathogens, and they are toxic to animals and humans and frequently detected in cereal crops [113]. Because of their diversity in structure and biological activity, trichothecenes are of concern in agriculture, food contamination, health care, and building protection.

Trichoderminol **311** was isolated from the filamentous fungus *Trichoderma albolutescens* [114]. Trichobreols A–E **312**–**316** were isolated from the marine-derived fungus *Trichoderma* cf. *brevicompactum* [115,116]. Trichobreols A–E **312**–**316** inhibited the growth of two yeast-like fungi, *Candida albicans*, and *Cryptococcus neoformans*, with a range of MIC values of 1.6 to 50 μg/mL [115,116]. Three new macrocyclic trichothecenes, miophytocen D **317**, roridin F **318**, and satratoxin I **319**, were isolated from a deadly poisonous mushroom *Podostroma cornu-damae* [117]. Satratoxin I **319** showed cytotoxic potency to etoposide against four human breast cancer cell lines (Bt549, HCC70, and MDA-MB-231), with IC_50_ values of 1.8, 7.7, and 3.6 μM, respectively.

Epiroridin acid **320**, verrucarins Y **321** and Z **322**, and dihydromyrothecine C **323**, four new macrocyclic trichothecenes, were isolated from the endophytic fungus *Myrothecium roridum* [118,119,120,121]. The cytotoxic mechanisms result showed that the epiroridin acid **320** induced the apoptosis of cancer cell HepG-2 via activation of caspase-9 and caspase-3, up-regulation of *bax* gene expression, down-regulation of *bcl*-2 gene expression, and disruption of the mitochondrial membrane potential of the HepG-2 cell [118].

Chartarenes A–D **324**–**327** were isolated from the sponge-derived fungus *Stachybotrys chartarum* [122]. Chartarenes A–D **324**–**327** exerted potent or selective inhibition against a panel of tumor cell lines including HCT-116, HepG2, BGC-823, NCI-H1650, and A2780, with IC_50_ values ranging from 0.68 to 10 µM. In addition, chartarenes B **326**, C **327**, and D **324** showed potent inhibition against tumor-related kinases FGFR3, IGF1R, PDGFRb, and TRKB, with IC_50_ values ranging from 0.1 to 12.9 µM.



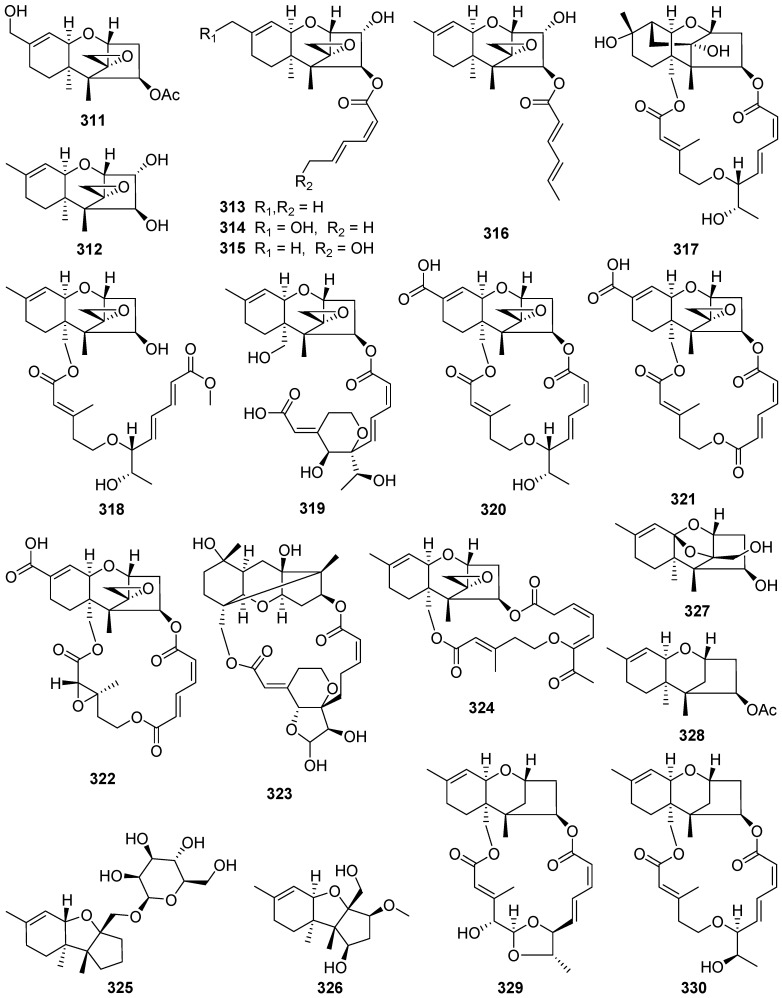



12-Deoxytrichodermin **328**, 12-deoxyroridin J **329**, and 12-deoxyepiisororidin E **330** were isolated from the fungus *Calcarisporium arbuscular*, and *Trichoderma* sp., respectively [123,124]. The structure-activity relationship investigation of **328**–**330** with other known natural trichothecenes against a human colon cancer cell line (COLO201) and filamentous fungus *Cochliobolus miyabeanus* revealed that the 12-epoxide functionality is essential for the antifungal activity [124].



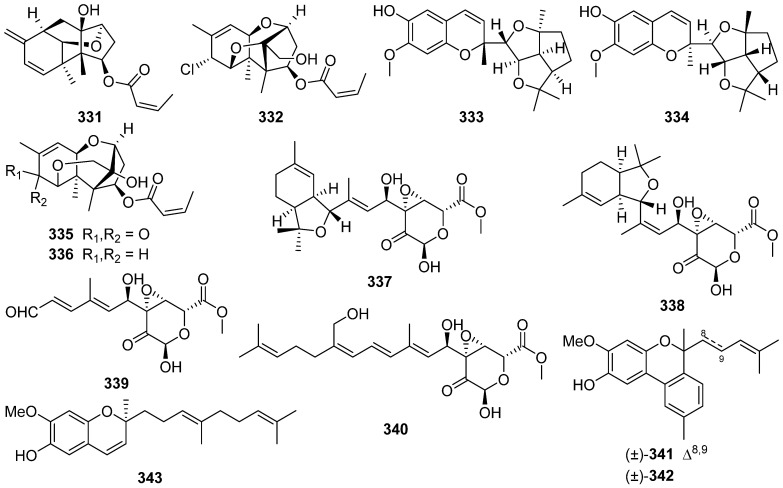



**Scheme 13:**
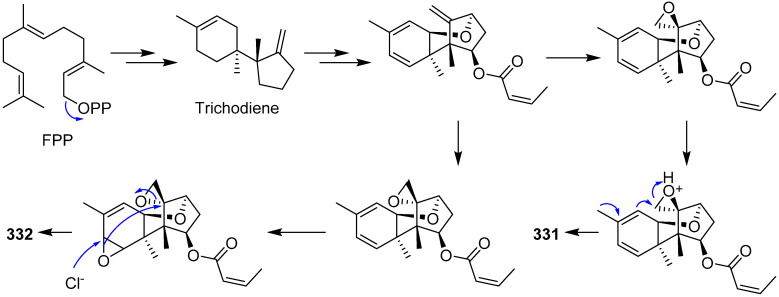
Proposed biosynthetic pathway for **331** and **332** (Reference [126]).

**Scheme 14:**
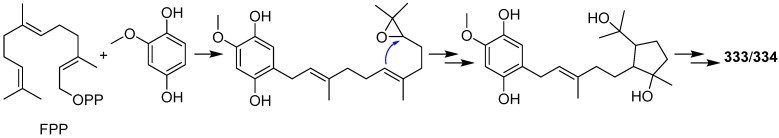
Proposed biosynthetic pathway for **333** and **334** (Reference [125]).

Four novel trichothecene sesquiterpenoids possessing new ring systems, trichothecrotocins A **331** and B **332**, trichothecrotocins K **335** and L **336**, and a merosesquiterpenoid racemate possesses a novel 6/6–5/5/5 fused ring system, (±)-trichothecrotocin C (**333** and **334**), and seven new merosesquiterpenoids, trichothecrotocins D–J **337**–**343**, were obtained from potato endophytic fungus *Trichothecium crotocinigenum* by bioguided isolation (Scheme 13 and Scheme 14) [125,126]. Compounds **337**–**340** were rare meroterpenoids featuring a *seco*-phenyl group, while **337** and **338** possessed a novel 6–6/5 fused ring system. Compounds **331**–**335**, and **337**–**340** showed antiphytopathogenic activities with MIC values of 8–128 μg/mL [125,126].

**Scheme 15:**
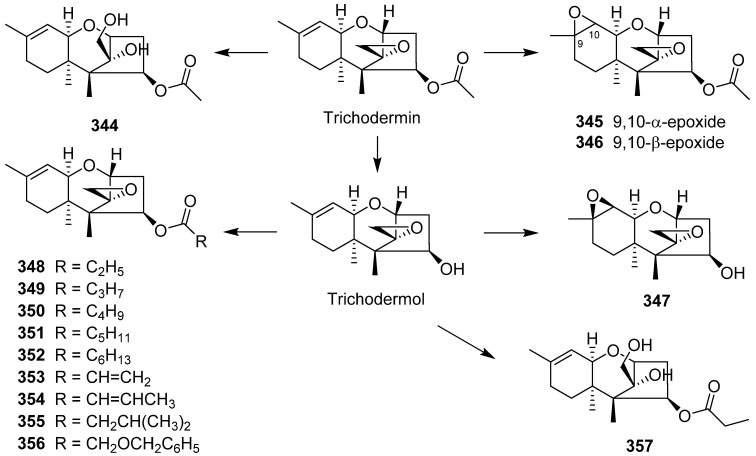
Chemical transformations were carried out on trichodermin and trichodermol for the preparation of compounds **344**–**357** (Reference [127]).

The semisynthesis of several trichodermin and trichodermol derivatives has been developed (Scheme 15) [127]. Some derivatives with a short chain at the C-4 position displayed selective antimicrobial activity against *Candida albicans* and they showed MIC values similar to those displayed by trichodermin. It was important to highlight the cytotoxic selectivity observed for compounds **350**, **354**, and **356**, which presented average IC_50_ values of 2 µg/mL and were cytotoxic against tumorigenic cell line MCF-7 (breast carcinoma) and not against Fa2N4 (non-tumoral immortalized human hepatocytes).

Three novel highly oxygenated *α*-pyrone merosesquiterpenoids, emerones A–C **358**–**360**, have been obtained from the fungus *Emericella* sp. XL029 [128]. Structurally, emerone A **358** possessed an unprecedented 5/7 bicyclic ring architecture, emerone B **359** had an unusual substituted 10-membered ring, and emerone C **360** had an undescribed norsesquiterpene skeleton. Ochraceopone F **361**, a new α-pyrone merosesquiterpenoid possessing an angular tetracyclic carbon skeleton, was isolated from the marine fungus *Aspergillus flocculosus* [129]. 

Five new highly oxygenated *α*-pyrone merosesquiterpenoids, ochraceopones A–E **362**–**366**, were isolated from an Antarctic soil-derived fungus *Aspergillus ochraceopetaliformis* [130]. Ochraceopones A–D **363**–**366** were the first examples of *α*-pyrone merosesquiterpenoids possessing a linear tetracyclic carbon skeleton. Ochraceopone A **363** exhibited antiviral activities against the H3N2 influenza virus with IC_50_ values of 12.2 μM. Yaminterritrem C **367** was isolated from a deep-sea-derived fungus *Penicillium chrysogenum* [131]. Verruculides A **368** and B **369** were isolated from a culture broth of the Indonesian ascidian-derived *Penicillium verruculosum* [132]. Verruculide A **368** inhibited the activity of PTP1B with an IC_50_ value of 8.4 μM.



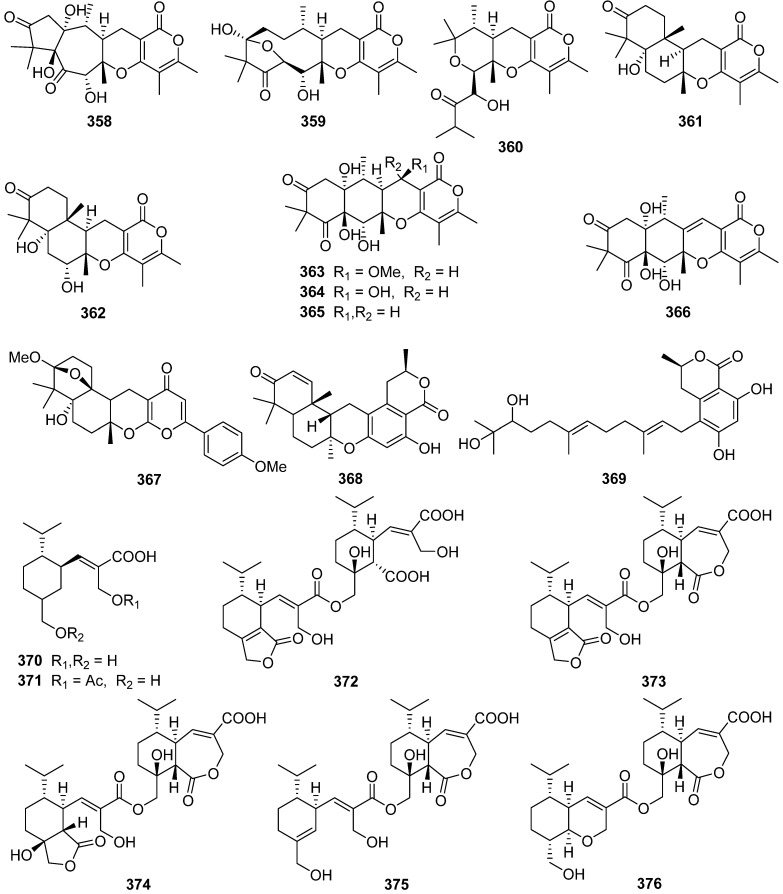



**Scheme 16:**
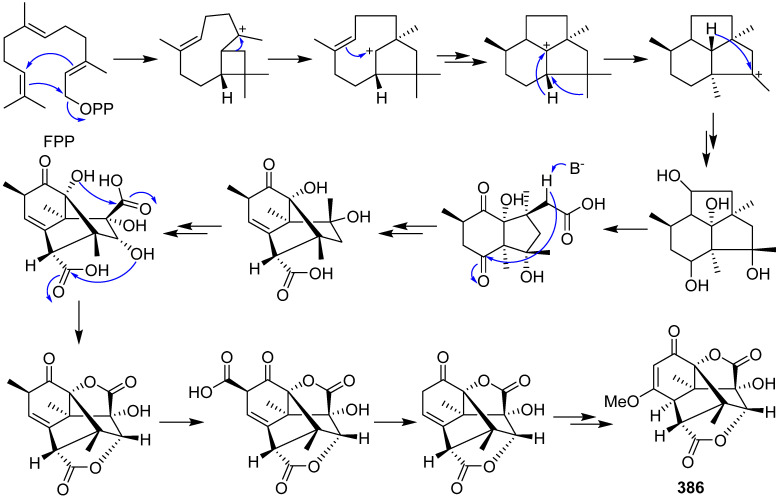
Proposed biosynthetic pathway for (+)-preuisolactone A **386** (Reference [135]).

Two new sesquiterpenes **370** and **371** and seven new dimeric norsesquiterpene congeners, divirensols A–G **372**–**378**, along with seven new firstin-class trimeric sesquiterpenes, trivirensols A–G **379**–**385**, were obtained from the Australian termite nest-derived fungus *Trichoderma virens* [133,134]. A pair of rare naturally enantiomeric norsesquiterpenoids, (±)-preuisolactone A (**386** and **387**) featuring an unprecedented tricyclo[4.4.0^1,6^.0^2,8^]decane carbon scaffold were isolated from *Preussia isomera* (plausible biosynthetic pathway shown in Scheme 16) [135]. (±)-Preuisolactone A (**386** and **387**) exhibited remarkable antibacterial activity against *Micrococcus luteus* with a MIC value of 10.2 μM.



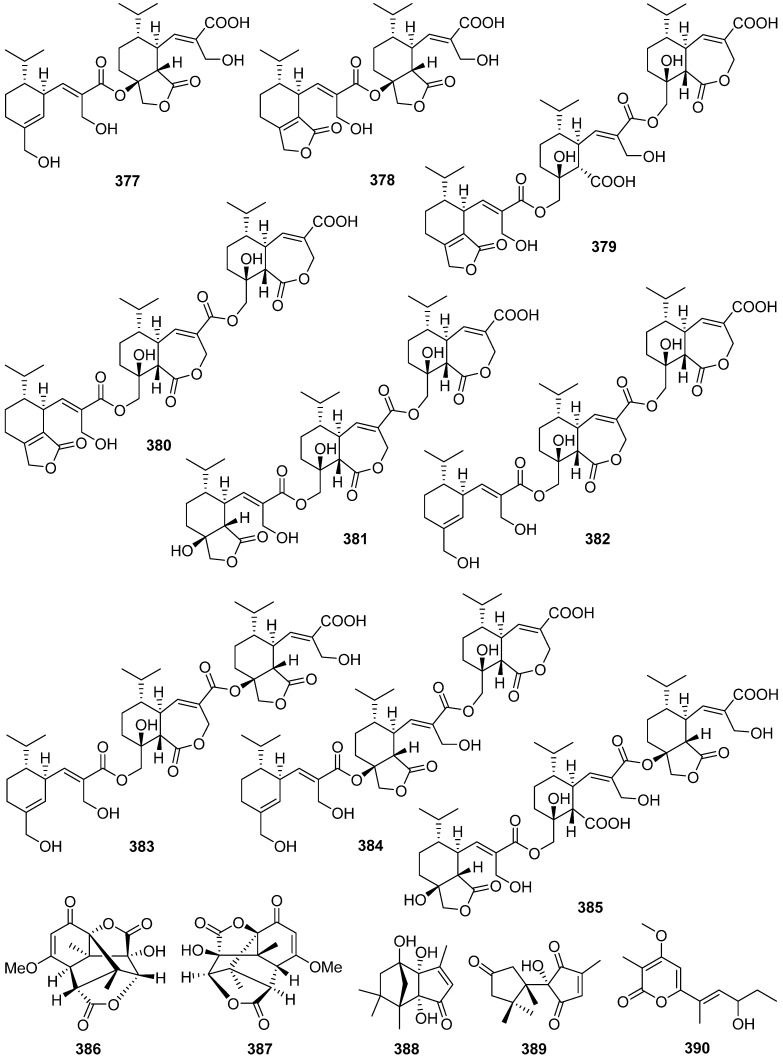



**Scheme 17:**
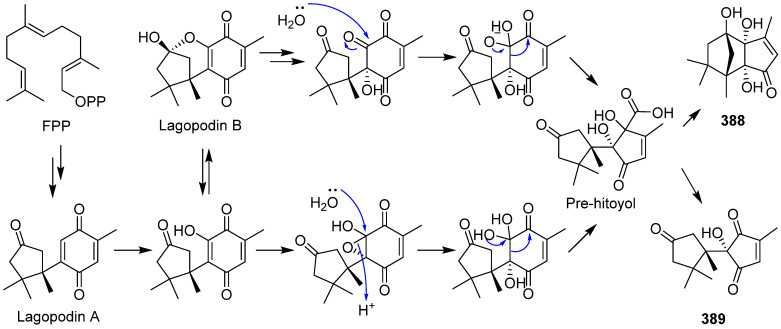
Plausible biosynthetic pathway for hitoyol A **388** and hitoyol B **389** (Reference [136]).

Hitoyol A **388**, an unprecedented norsesquiterpenoid with an exo-tricyclo[5.2.1.0^2,6^]decane skeleton, along with a novel skeletal hitoyol B **389** containing 4-cyclopentene-1,3dione, was isolated from the fungus *Coprinopsis cinerea* [136]. Hitoyol A **388** was possibly biosynthesized through decarboxylation-induced cyclization of lagopodin B, a known cuparene-type sesquiterpenoid (Scheme 17). Hitoyol B **389** showed weak antimalarial activity against *Plasmodium falciparum* with an IC_50_ of 59 μM.

An *α*-pyrone 9-hydroxyxylarone **390** was isolated from a culture broth of endophytic fungus *Xylaria* sp. NC1214 [137]. Four new polyenic *α*-pyrone mycotoxins, avertoxins A–D **391**–**394**, were obtained from an endophytic fungus *Aspergillus versicolor* [138]. Avertoxins B **392** and C **393** showed activity against human tumor HCT116 and HeLa cell lines with an IC_50_ value of 10 μM. And avertoxin B **392** was an active inhibitor against human acetylcholinesterase with the IC_50_ value of 14.9 μM.



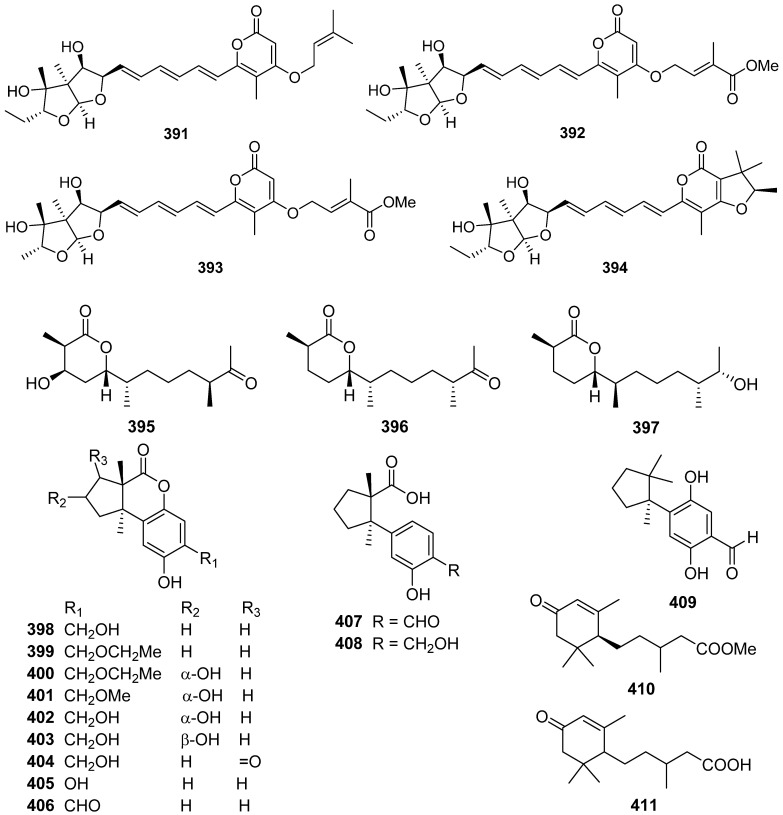



### 2.10. Other Types

Three new sesquiterpenoids, chermesiterpenoids A–C **395**–**397**, were isolated and identified from the marine red algal-derived fungus *Penicillium chermesinum* [139]. Chermesiterpenoid B **396** showed antimicrobial activities against the aquatic pathogens *Vibrio anguillarum*, *Vibrio parahaemolyticus*, *Micrococcus luteus*, and human pathogen *Escherichia coli* with minimum inhibitory concentration (MIC) values of 0.5, 16, 64, and 64 µg/mL, respectively. Similarly, chermesiterpenoid C **397** showed activities against the aquatic pathogens *V. anguillarum*, *V. parahaemolyticus*, and *M. luteus* with MIC values of 1, 32, and 64 µg/mL, respectively. Chermesiterpenoids A–C **395**–**397** exhibited activity against the plant pathogenic fungus *Colletottichum gloeosporioides* with MIC values of 64, 32, and 16 µg/mL, respectively.



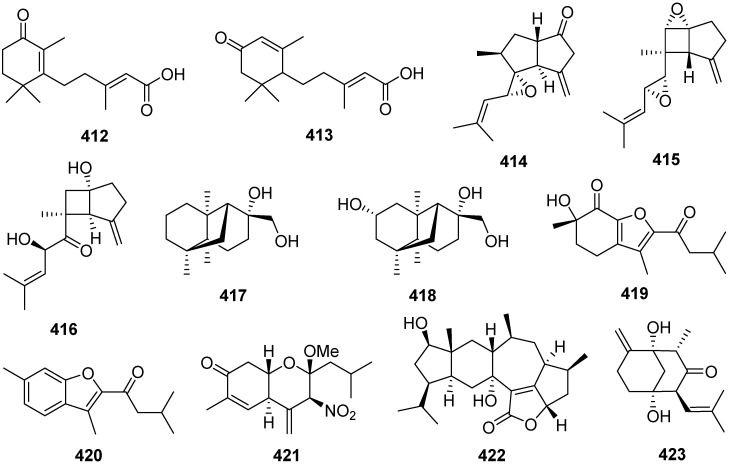



Fomitopins A–L **398**–**409** were isolated via bioassay-guided purification from the bracket fungus *Fomitopsis pinicola* [140]. Fomitopin K **408** exhibited the most potent anti-inflammatory activity with IC_50_ of 0.81 μM for inhibition of superoxide anion generation and IC_50_ of 0.74 μM for inhibition of elastase release. Fomitopins J **407** and L **409** also exhibited moderate inhibition of superoxide anion generation with IC_50_ of 1.66 and 1.72 μM, respectively.

1-Methoxypestabacillin B **410** was obtained from the solid cultures of a mangrove endophytic fungus *Diaporthe* sp. SCSIO 41011 [141]. Pestabacillin B **411** was isolated from the co-culture of the endophytic fungus *Pestalotiopsis* sp. with *Bacillus subtilis* [19]. Two new abscisic acid-type sesquiterpenes **412** and **413** were isolated from the fermentation extract of *Amycolatopsis alba* [142]. Pseudapenes A–C **414**–**416** possessing unique carbon skeletons were isolated from the marine-derived fungus *Pseudallescheria apiosperma* [143].

**Scheme 18:**
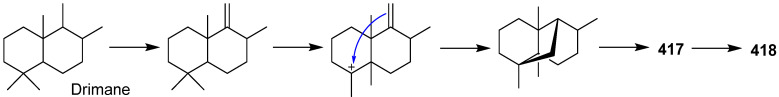
The proposed formation of **417** and **418** from the drimane-type sesquiterpenoid skeleton (Reference [144]).

Emericellins A **417** and B **418**, representing a new type of sesquiterpenoid with an unprecedented tricyclo[1,2,4,4]hendecane scaffold (Scheme 18), were isolated from the liquid cultures of an endophytic fungus *Emericella* sp. associated with the leaves of *Panax notoginseng* [144]. Emericellins A **417** and B **418** displayed moderate activities against three fungal strains (*Verticillium dahliae* Kleb, *Helminthosporium maydis*, and *Botryosphaeria dothidea*) and three bacterial strains (*Bacillus subtilis*, *Bacillus cereus,* and *Escherichia coli*) with MIC values of 25–50 μg/mL.

Stereumenes A–C **419**–**421** were isolated and identified from the fungus *Stereum* sp. [145]. Stereumene B **420** showed weak nematicidal activity against *Caenorhabditis elegans*, which killed 41.1% of *C. elegans* at 200 mg/L in 24 h. Sesteralterin **422** was obtained from the culture extract of an *Alternaria alternata* strain isolated from the surface of the marine red alga *Lomentaria hakodatensis* [146]. Colletotrichine A **423** was obtained from the endophyte fungus *Colletotrichum gloeosporioides* [147].

Four novel mixed terpenes, stereumamides A–D **424**–**427**, which were sesquiterpenes combined with α-amino acids to form quaternary ammonium hybrids, were isolated from the mycelium of mushroom *Stereum hirsutum* [148]. Stereumamides A **424** and D **427** showed antibacterial activity against *Escherichia coli*, *Staphylococcus aureus*, and *Salmonella typhimurium*, with MIC values of 12.5–25.0 μg/mL.



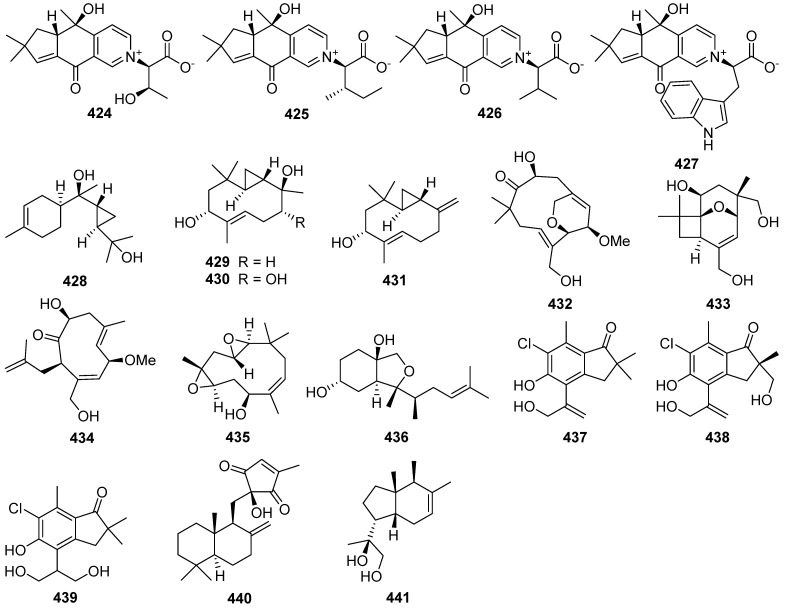



**Scheme 19:**
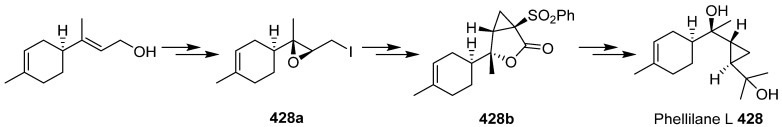
Total synthesis of phellilane L **428** (Reference [149]).

Phellilane L **428**, a new cyclopropane-containing sesquiterpenoid, was isolated from the medicinal mushroom *Phellinus linteus* [149]. The first asymmetric, protecting group-free total synthesis of the sesquiterpenoid phellilane L **428**, featuring a highly stereoselective one-pot synthesis involving intermolecular alkylation/cyclization/lactonization on epoxyiodide **428a** to construct the key cyclopropane-γ-lactone intermediate **428b** has been reported (Scheme 19) [149].

Hypocoprins A–C **429**–**431** have a distinctive ring system consisting of fused cyclopropane and cyclodecene units were isolated from the Coprophilous fungus *Hypocopra rostrate* [150]. Pestaloporonins A–C **432**–**434**, three new sesquiterpenoids related to the caryophyllene-derived punctaporonins, were isolated from cultures of a fungicolous isolate of *Pestalotiopsis* sp. MYC-709 [151]. Among them, pestaloporonins A **432** and B **433** contained new bicyclic and tricyclic ring systems, respectively, and the absolute configuration of **432** was confirmed by single-crystal X-ray crystallographic analysis.

**Scheme 20:**
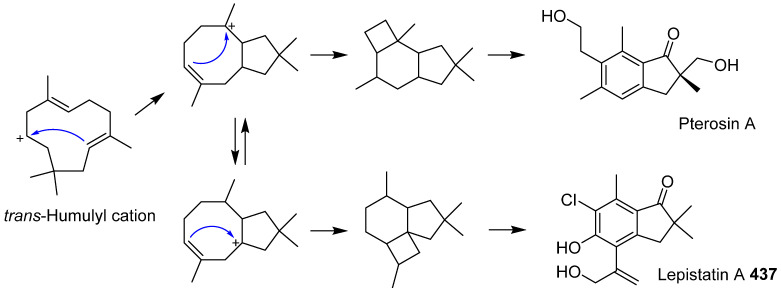
Plausible biosynthetic pathway for lepistatin A **437** and pterosin A (Reference [154]).

Phomanoxide **435** was isolated from the solid substrate fermentation cultures of the fungus *Phoma* sp. [152]. Colletotrichine B **436** was produced by the fungal *Colletotrichum gloeosporioides* [153]. Three new chlorinated sesquiterpenes, lepistatins A–C **437**–**439**, were isolated from the culture broth of Basidiomycete *Lepista sordida* [154]. The structures of lepistatins A–C **437**–**439** feature the indanone core structure but differ from other indanone-containing sesquiterpenes of fungal origin by the alkyl substitution pattern. This indicates that lepistatins A–C **437**–**439** probably possessed a new sesquiterpene scaffold derived from the common precursor, trans-humulyl cation, by an alternative cyclization (Scheme 20).

**Scheme 21:**
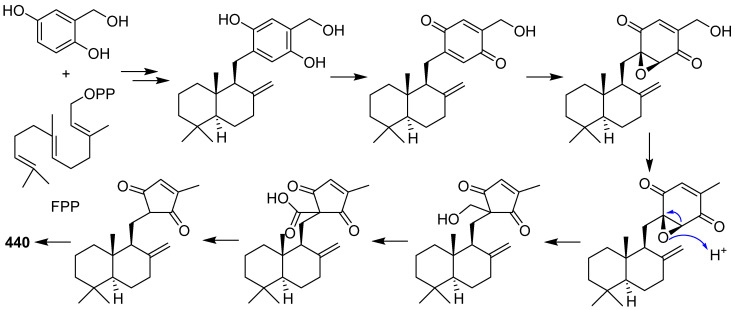
Plausible biosynthetic pathway of penicilliumin B **440** (Reference [155]).

A novel sesquiterpene methylcyclopentenedione, penicilliumin B **440**, was obtained from a deep sea-derived fungus *Penicillium* sp. F00120 [155]. Penicilliumin B **440**, presenting the first example with the sesquiterpene cyclopentenedione skeleton as natural products (Scheme 21), was structurally determined by analysis of the NMR and MS spectroscopic data, while the absolute configurations were assigned by single-crystal X-ray experiments. Penicilliumin B **440** with low toxicity showed significant potential to inhibit the kidney fibrogenic action in vitro by a mechanism dependent on disruption of oxidative stress. Seiricardine D **441** was a new bicyclic sesquiterpene obtained from the endophytic fungus *Cytospora* sp. [156]. Twenty new sesquiterpenes (**442**–**461**) were isolated from the endophytic fungus *Pseudolagarobasidium acaciico* [157]. Among them, compounds **459** and **460** displayed cytotoxicity against several cancer and normal cell lines.



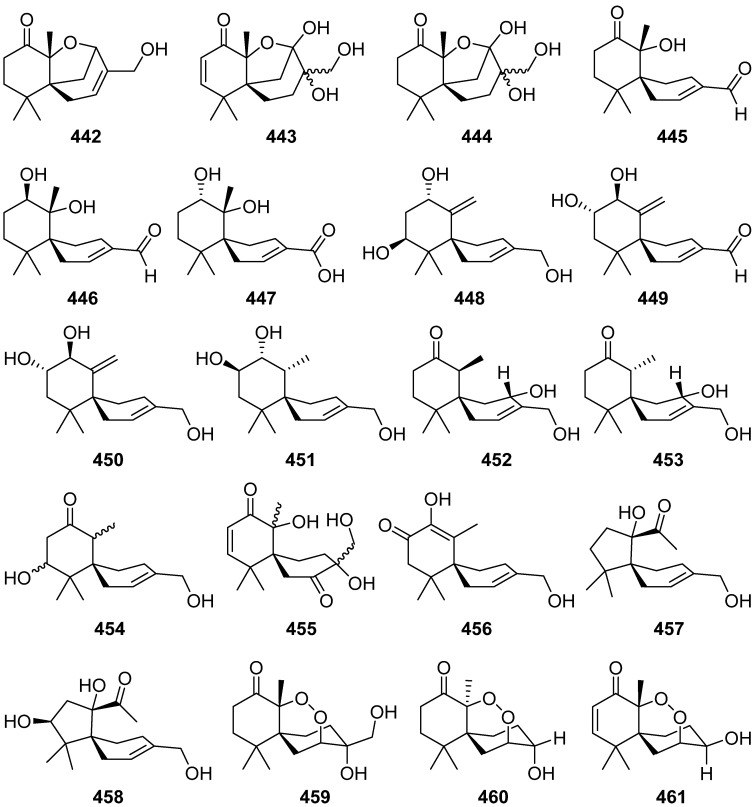



## 3. Biosynthesis

### 3.1. Asperterpenoid A

**Scheme 22:**
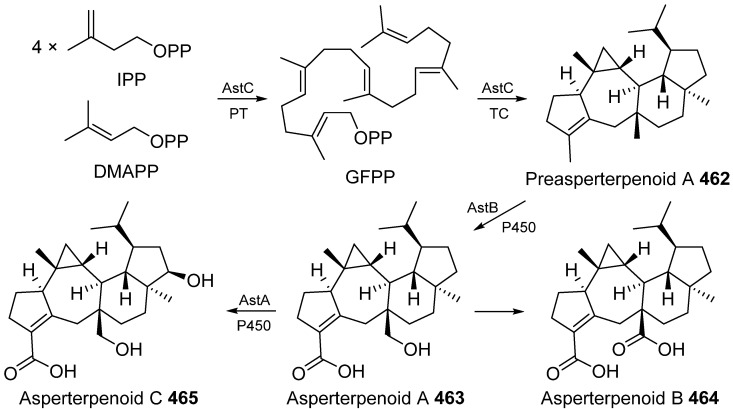
Complete biosynthetic pathway of asperterpenoids A–C **463**–**465** (Reference [158]).

A putative three-gene cluster for asperterpenoid A was identified [158]. Stepwise reconstitution of this gene cluster in *Aspergillus oryzae* reveals that *astC* encodes a sesterterpene cyclase to synthesize preasperterpenoid A **462**, which was dually oxidized by a P450 enzyme AstB to give asperterpenoid A **463** along with a minor product asperterpenoid B **464**, and asperterpenoid A **463** was further oxidized by another P450 enzyme AstA to afford a new sesterterpenoid asperterpenoid C **465** (Scheme 22). Asperterpenoids A **463** and B **464** exhibit potent inhibitory activity against *Mycobacterium tuberculosis* protein tyrosine phosphatase B with IC_50_ values of 3–6 μM.

### 3.2. Cuparene

**Scheme 23:**
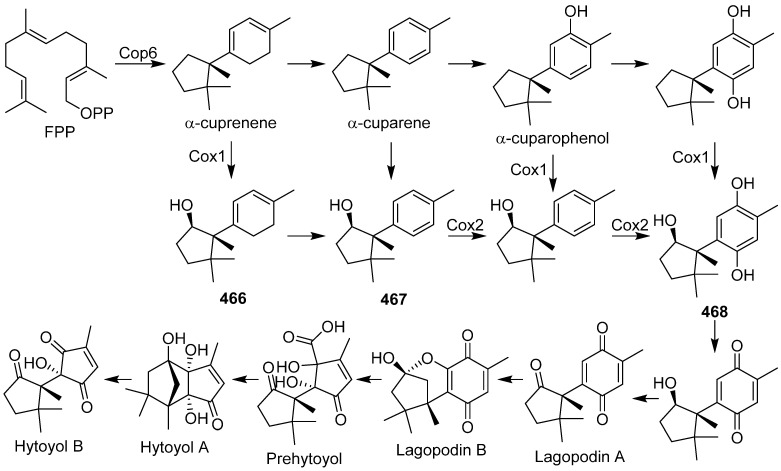
The proposed biosynthetic pathway of lagopodins and hytoyols (Reference [159]).

Use of the *ku*70-deficient strain of *Coprinopsis cinerea* enabled confirmation within the native context of the central role the sesquiterpene synthase Cop6 plays in lagopodin biosynthesis [159]. Furthermore, yeast in vivo bioconversion and in vitro assays of two cytochrome P450 monooxygenases Cox1 and Cox2 allowed elucidation of the network of oxidation steps that build structural complexity onto the *α*-cuprenene framework during the biosynthesis of lagopodins (Scheme 23). Three new compounds **466**–**468** were identified as intermediates formed by the redox enzymes.

### 3.3. Fusariumdiene and Fusagramineol

**Scheme 24:**
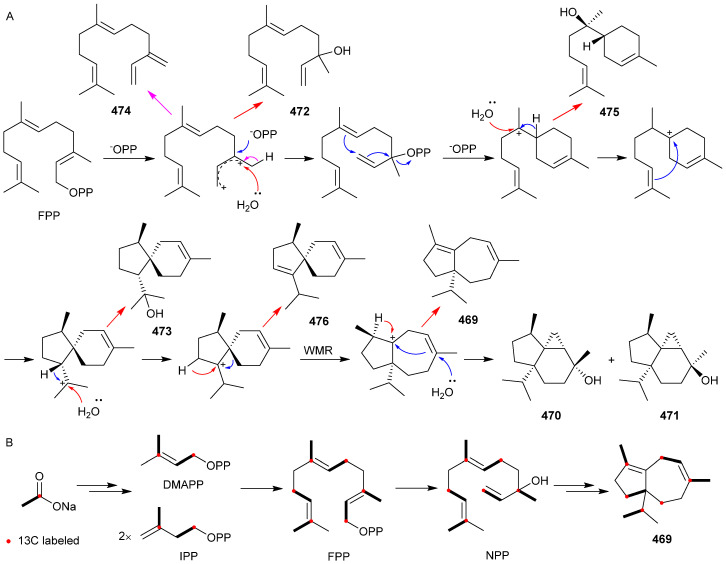
(**A**) Proposed mechanisms for the enzymatic cyclization of FPP to compounds **469**–**476**; (**B**) Summary of feeding experiments with [1-^13^C,^2^H_3_] sodium acetate (Reference [160]).

The novel sesquiterpenes fusariumdiene **469**, *epi*-fusagramineol **470**, and fusagramineol **471** with 5/7 bicyclic and 5/6/3 tricyclic ring systems, respectively, as well as five known sesquiterpenes **472**–**476** have been produced by exploiting the potential power of sesquiterpene synthase FgJ03939 from *Fusarium graminearum* in a farnesyl diphosphate-overexpressing *Saccharomyces cerevisiae* chassis (Scheme 24) [160].

### 3.4. Hirsutenoid

**Scheme 25:**
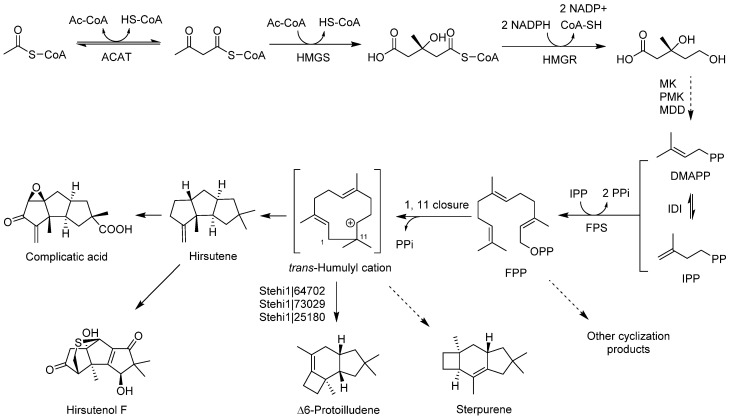
Overview of pathways to *trans*-humulyl cation-derived sesquiterpenoids in *S. hirsutum* (Reference [161]).

The identification and successful cloning of the previously elusive hirsutene synthase from the wood-rotting mushroom *Stereum hirsutum* provide the biosynthetic pathways of hirsutane-type sesquiterpenoids (Scheme 25) [161]. The hirsutene synthase, as an unexpected fusion protein of a sesquiterpene synthase (STS) with a C-terminal 3-hydroxy-3-methylglutaryl-coenzyme A (3-hydroxy-3-methylglutaryl-CoA) synthase (HMGS) domain, was part of a biosynthetic gene cluster that includes P450s and oxidases that were expressed and could be cloned from cDNA.

### 3.5. Koraidiol

**Scheme 26:**
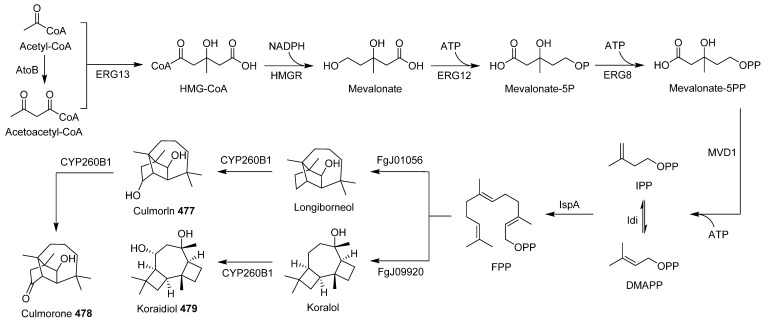
Biosynthesis pathways of culmorin **477**, culmorone **478**, and koraidiol **479** in *E. coli* (Reference [162]).

Two known oxygenated sesquiterpenoid products, culmorin **477** and culmorone **478**, and a new compound, koraidiol **479**, were successfully generated and characterized by a combinatorial biosynthesis approach which was utilized by the combination of a promiscuous myxobacterial P450 (CYP260B1) with two sesquiterpene cyclases (FgJ01056, FgJ09920) of filamentous fungi *Fusarium graminearum* (Scheme 26) [162].

### 3.6. Protoilludenes

**Scheme 27:**
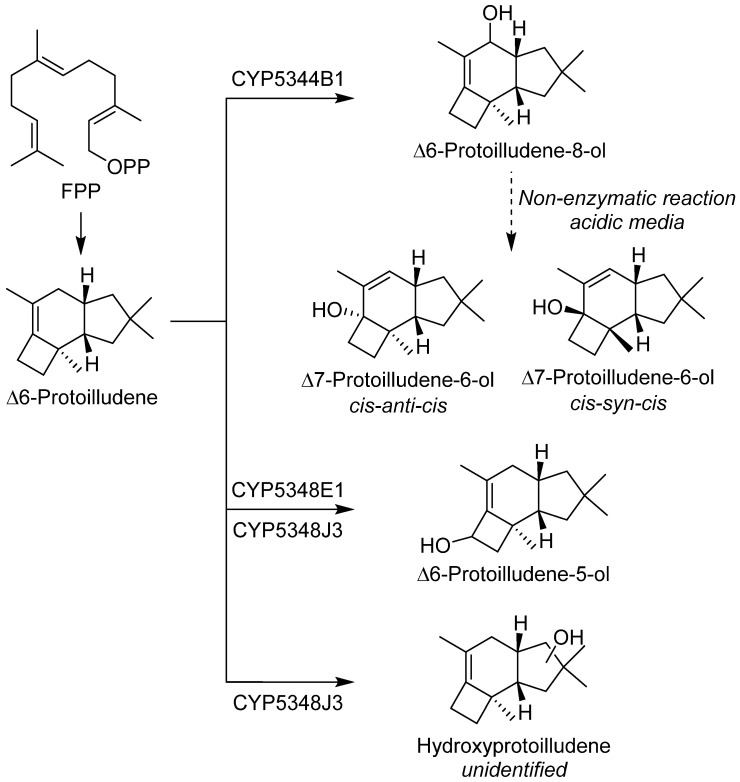
Reaction pathways of protoilludene metabolism by PpSTS-08 and PpCYPs (Reference [163]).

Sixteen sesquiterpene synthases genes as full-length cDNAs have been isolated by using RT-PCR, and heterologous expression revealed that the sesquiterpene synthases could produce a series of sesquiterpene scaffolds with distinct metabolic profiles (Scheme 27) [163].

### 3.7. Trichothecenes

**Scheme 28:**
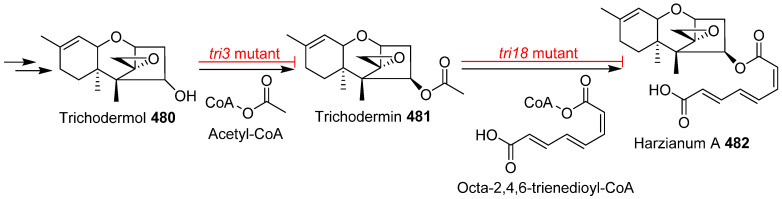
Biosynthesis pathways of trichodermol **480** to harzianum A **482**. The symbol ⊥ indicates that the pathway is partially or completely blocked at the step indicated in the *tri3* and *tri18* mutants (Reference [164]).

The acyltransferase-encoding gene *tri18*-encoded acyltransferase (TRI18) and a previously characterized acyltransferase (TRI3) were required in the saprotroph *Trichoderma arundinaceum* for conversion of the trichothecene biosynthetic intermediate trichodermol **480** to harzianum A **482**, an antifungal trichothecene analog with an octa-2,4,6-trienedioyl acyl group [164]. Previous studies indicate that *tri18* may not be necessary for the biosynthesis of harzianum A **482** because all catalytic activities required for its formation can be accounted for by activities of enzymes (TRI5, TRI4, TRI22, TRI17, and TRI3) encoded by other *tri* genes [165,166]. Further analysis proposed that TRI3 catalyzes trichothecene 4-O-acetylation, and subsequently, TRI18 catalyzes replacement of the resulting acetyl group with octa-2,4,6-trienedioyl to form harzianum A **482** (Scheme 28) [164].

**Scheme 29:**
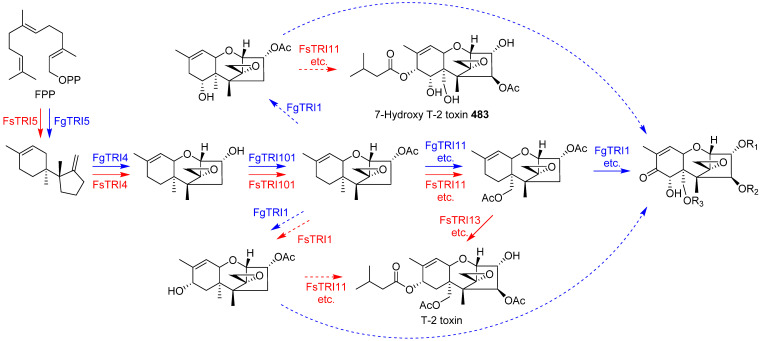
Biosynthetic approach to generate 7-hydroxy T-2 toxin **483.** Red arrows indicate the metabolic pathway of *F. sporotrichioides*, whereas blue arrows indicate that of *F. graminearum*. Solid arrows indicate the main pathway, and dotted arrows indicate the shunt pathway (Reference [167]).

An artificial metabolic route to an unnatural trichothecene was designed by taking advantage of the broad substrate specificities of the T-2 toxin biosynthetic enzymes of *Fusarium sporotrichioides* [167]. By feeding 7-hydroxyisotrichodermin, a shunt pathway metabolite of *F. graminearum*, to a trichodiene synthase-deficient mutant of *F. sporotrichioides*, 7-hydroxy T-2 toxin **483** was obtained as the final metabolite (Scheme 29). The toxicity of 7-hydroxy T-2 toxin **483** was 10 times lower than that of T-2 toxin in HL-60 cells.

**Scheme 30:**
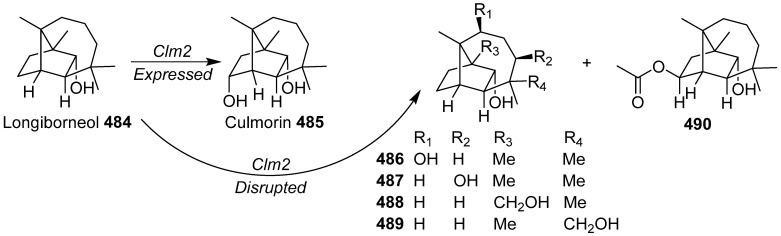
Biosynthetic pathways of **484**–**490** (Reference [168]).

The candidate gene, *Clm*2, a second structural gene required for culmorin biosynthesis in the plant pathogen *Fusarium graminearum*, encodes a regio- and stereoselective cytochrome P450 monooxygenase for C-11 of longiborneol **484** (Scheme 30) [168]. *Clm*2 gene disruptants were grown in liquid culture and assessed for culmorin production via HPLC-evaporative light scattering detection. The analysis indicated a complete loss of culmorin **485** from the liquid culture of the Δ*Clm*2 mutants. Culmorin production resumed in a Δ*Clm*2 complementation experiment. A detailed analysis of the secondary metabolites extracted from the largescale liquid culture of disruptant Δ*Clm*2D20 revealed five new natural products: **486**–**490**. The structures of the new compounds were elucidated by a combination of HRMS, 1D and 2D NMR, and single-crystal X-ray crystallography analysis.

## 4. Conclusions and Future Prospects

Natural products, in particular bioactive molecules as precursor pharmaceutical compounds, have attracted particular attention in the field of health promotion and drug discovery and development. Compared with other sources, fungal species play a decisive role in bio-transformations and drug synthesis owing to their wide varieties, easy cultivation, diverse chemical compositions, and distinct biological activities. This process has been accelerated by considerable advances in microbial genome research and in understanding the structure of genes and their corresponding products. Genome mining-based natural products discovery programs mainly use the most identifiable terpene synthases and prenyltransferases to locate and quickly identify new terpenoids. In the last five years, nearly 500 new sesquiterpenes, including about 20 new skeletons were identified from fungi. These sesquiterpenoids exhibit various biological activities, such as anti-tumor, anti-viral, anti-microbial, anti-inflammatory, etc. These efforts have clearly led to a global promotion of discovery and characterization of fungal terpenoids and offer optimism for the future of fungal terpenoid discovery.

This review summarized the isolation, chemical structures, plausible biosynthetic pathways, bioactivity, chemical synthesis, and biosynthesis of 490 recent sesquiterpenoids. This could be a useful reference for modern researchers studying this category of compounds.

## Data Availability

Not applicable.
